# Unveiling the “hidden quality” of the walnut pellicle: a precious source of bioactive lipids

**DOI:** 10.3389/fpls.2024.1395543

**Published:** 2024-06-18

**Authors:** Ramona Abbattista, Noah G. Feinberg, Isabel F. Snodgrass, John W. Newman, Abhaya M. Dandekar

**Affiliations:** ^1^ Department of Plant Sciences, University of California, Davis, Davis, CA, United States; ^2^ Western Human Nutrition Research Center, United States Department of Agriculture, Davis, CA, United States; ^3^ West Coast Metabolomics Center, Genome Center, University of California, Davis, Davis, CA, United States; ^4^ Department of Nutrition, University of California, Davis, Davis, CA, United States

**Keywords:** walnut, tree nuts, seed coat, pellicle, waste by-products, metabolomics, bioactive lipids

## Abstract

Tree nut consumption has been widely associated with various health benefits, with walnuts, in particular, being linked with improved cardiovascular and neurological health. These benefits have been attributed to walnuts’ vast array of phenolic antioxidants and abundant polyunsaturated fatty acids. However, recent studies have revealed unexpected clinical outcomes related to walnut consumption, which cannot be explained simply with the aforementioned molecular hallmarks. With the goal of discovering potential molecular sources of these unexplained clinical outcomes, an exploratory untargeted metabolomics analysis of the isolated walnut pellicle was conducted. This analysis revealed a myriad of unusual lipids, including oxylipins and endocannabinoids. These lipid classes, which are likely present in the pellicle to enhance the seeds’ defenses due to their antimicrobial properties, also have known potent bioactivities as mammalian signaling molecules and homeostatic regulators. Given the potential value of this tissue for human health, with respect to its “bioactive” lipid fraction, we sought to quantify the amounts of these compounds in pellicle-enriched waste by-products of mechanized walnut processing in California. An impressive repertoire of these compounds was revealed in these matrices, and in notably significant concentrations. This discovery establishes these low-value agriculture wastes promising candidates for valorization and translation into high-value, health-promoting products; as these molecules represent a potential explanation for the unexpected clinical outcomes of walnut consumption. This “hidden quality” of the walnut pellicle may encourage further consumption of walnuts, and walnut industries may benefit from a revaluation of abundant pellicle-enriched waste streams, leading to increased sustainability and profitability through waste upcycling.

## 1 Introduction

Consumer demand for tree nuts, such as almonds, pistachios, and walnuts, continues to increase globally. Based on available data from the USDA Economic Research Service, the per capita consumption of shelled tree nuts has increased from 3.8 pounds in 2010 to almost 6 pounds in 2021. Shelled tree nuts, which consist of only the edible portion of the nut, are popular among consumers for various reasons. They have cultural significance, can be utilized in many diverse culinary applications, are a tasty, nutritional, and convenient snack, and are increasingly recognized for their health benefits. Government health agencies worldwide encourage the consumption of tree nuts, describing them as nutrient-dense foods that provide essential dietary components and bioactive compounds^1^. The health-promoting compounds rich in tree nuts, typically lacking in Western diets, are associated with lowering cardiovascular disease mortality, diabetes, and cancer risk (Ros et al., 2021).

Regarding walnuts specifically, their worldwide consumption experienced the same growth as the broader group of tree nuts in the last decade. The expansion of walnut industries across the globe to meet consumer demand resulted in a total industry value of $7.53B in 2022. The global industry is projected to grow at a rate of 5.15% between 2023 and 2029, reaching an approximate value of $10.71B before the turn of the next decade^2^. Walnuts continue to be enjoyed by consumers due to their unique taste, texture, and aroma, as well as their convenience as a highly nutritious snack food. Furthermore, incisive marketing has utilized health research outcomes and institutional health claims to drive consumer interest based on the associated health benefits of walnut consumption (Lockyer et al., 2022). Consumption of 43 g of walnuts daily is supported by the Food and Drug Administration (FDA) for reducing the risk of coronary heart disease^3^, and 30 g per day by the EU and UK for improving vascular function (Great Britain nutrition and health claims (NHC) register, 2022)^4^. Other benefits associated with daily walnut consumption include reducing the risk of certain high-mortality-rate cancers, regulating metabolic dysfunction conditions such as diabetes and obesity, and supporting cognitive function and gut health (Jackson and Hu, 2014; Aune et al., 2016; Rock et al., 2017; Gorji et al., 2018; Fang et al., 2020; Pahlavani et al., 2020; Cahoon et al., 2021; Fitzgerald et al., 2021; Liu et al., 2021). A systematic review of cohort studies and randomized controlled trials published from 2017 to 2021 indicates the consistent association of walnut consumption with positive public health outcomes, specifically, improvement of blood lipid profiles and reduced cardiovascular disease (CVD) (Lockyer et al., 2022). A relatively short-term intake of walnuts was shown to change the blood lipid profile and, in particular, the metabolism of omega-3 alpha-linolenic (ALA), omega-6 linoleic (LA), eicosapentaenoic (EPA), and docosapentaenoic (DHA) acids; resulting in improved microvascular function (Holt et al., 2015).

While further evidence for these benefits is accumulating in other areas of ongoing health research, more work is needed to draw firm conclusions about the benefits of walnut consumption on health. This is mainly because the molecular mechanisms of these health outcomes still need to be completely understood or fully discovered. Compositional studies of walnut kernels, comprised of the meat (cotyledons and embryo) and the seed coat (pellicle or testa), have revealed each to be rich sources of bioactive compounds: essential lipids and potent, antioxidant phenolics, respectively (Vinson and Cai, 2012; Okatan et al., 2022).

Among the tree nuts, walnut meat is particularly rich in polyunsaturated fatty acids (PUFAs), in particular, alpha-linolenic acid (ALA; 18:3, n-3) and linoleic acid (LA; 18:2, n-6) (Jardim et al., 2023). The replacement of dietary saturated fats with unsaturated fats, especially omega-3 fatty acids (ω3FAs) like ALA, is connected to tangible health benefits. The reduction of low-density-lipoprotein cholesterol (LDL-C), mediated through the upregulation of lipo-protein-lipase (LPL), significantly decreases hypertriglyceridemia and the incidence of CVD (Hooper et al., 2020; Lockyer et al., 2022). PUFAs are also involved in cellular energetics by regulating cellular glucose and fatty acid metabolism. Interaction of PUFAs with peroxisome proliferator-activated receptors (PPARs) leads to transcriptional activation of genes involved in energy homeostasis, such as fatty acid β-oxidation, that support proper mitochondrial function (Magadum and Engel, 2018; Kytikova et al., 2020). Persistent mitochondrial dysfunction and disruption of energy homeostasis are linked to cardiovascular complications, as well as chronic inflammation and neurodegenerative conditions (Bhatti et al., 2017; Wang et al., 2019; Prasun, 2020; Amorim et al., 2022; Yang et al., 2022; Marchi et al., 2023). In addition to their essential role in energy metabolism, incorporating PUFAs into cellular membranes provides protective and stabilizing effects. This is crucial in high-fat-content tissues, such as nerves and the retina, where lack of ω3FAs has severe impacts on organ functionality, leading to neurodegenerative conditions (Alzheimer’s disease, dementia, etc.) and retinal deteriorative conditions, respectively (Sugasini et al., 2023; Wei et al., 2023). Additional mechanisms of action include modulation of inflammation status and resolution of local inflammation. Incorporation of ω3FAs into membranes displaces pro-inflammatory arachidonic acid (AA), as well as competes with AA for enzymatic production of autocoids (*i.e.*, signaling molecules acting as local hormones with brief duration to suppress inflammation), like eicosanoids (Ramsden et al., 2021). This mechanism operates alongside the activation of PPAR-gamma, which mediates inflammatory gene expression and NFkB activation to further resolve inflammation (Kim et al., 2023). Although these mechanisms of action have been extensively characterized, they cannot fully explain the broad spectrum of beneficial effects associated with walnut consumption, requiring further research.

Distinct from PUFA-rich walnut meat, the pellicle (seed coat), with its vast array of phenolic compounds, has received significant attention. Phenolic compounds (or phenolics), which encompass a large family of biomolecules containing a phenol structure, are thought to act synergistically with lipids to provide the health benefits associated with walnut consumption (Ni et al., 2022). Phenolic compounds are potent antioxidants used to mitigate oxidative stress, which, if left unmanaged, can result in oxidative damage to cellular components, resulting in reduced functionality. Oxidative damage is at the heart of many high-mortality conditions (particularly in the West); this includes heart disease, cancers, and chronic inflammation (Forman and Zhang, 2021). The walnut pellicle is especially abundant with phenolics, even when compared with other tree nuts, being significantly enriched in compounds from the hydrolysable tannin and flavonoid pathways. Some examples are gallic acid, ellagic acid, quercetin, and catechin, among many others (Acquaviva et al., 2021; Ni et al., 2022; Okatan et al., 2022). While this has been the primary focus of prior walnut pellicle research, the lipid fraction of the pellicle has yet to be thoroughly explored.

As with all plant tissues, the walnut pellicle comprises cells and thus contains ample lipids. These include the “standard” lipids, such as phospholipids (PLs), galactolipids, and triacylglycerols (TAGs), which are found in cellular membranes and lipid droplets for energy storage, together with fatty acids like palmitic acid (16:0), stearic acid (18:0), oleic acid (18:1Δ9), linoleic acid (18:2Δ9,12), and α-linolenic acid (18:3Δ9,12,15). Generally speaking, seed coats are known to accumulate many potent and unusual bioactive compounds to protect the seed within. Indeed, bioactive compounds have been discovered in the seed coat of other plant species (Ruiz et al., 2017; Havelt et al., 2019; Özdikicierler and Öztürk-Kerimoğlu, 2020; Dean, 2020; Commey et al., 2021; Alalwan et al., 2022) therefore this might extend to lipids. Lipid structures are diversified in plants by modulating carbon chain lengths, the number of unsaturation’s (double bonds) and their chain position, and the addition of carbon-chain modifications (i.e., hydroxylation, epoxidation, methylation, etc.), with countless combinations of these structural features. This population, therefore, represents a hidden fraction of “unusual lipids”, having peculiar structural features and lower abundance but with unique and often unexplored bioactivity for both plant and human health. Two of the best-understood classes of these “unusual lipids” are oxylipins and endocannabinoids because, in animals, they serve as the backbone of numerous signaling pathways and as homeostatic regulators for physiological processes. These include the resolution of inflammation and pain, activation of immune functions, and, via the gut-brain axis, improvement of cognitive health, mood, and behavioral disorders (Catani et al., 2018; Chiurchiù et al., 2018; Ledo et al., 2019; Baptista et al., 2020; Leuti et al., 2020; Duan et al., 2021; Kurano et al., 2022). Because of this critical functionality and to distinguish them from standard, more structural lipids, these unusual lipids are termed “bioactive”. In animals, these bioactive lipids are synthesized endogenously from common lipids, like PUFAs, in small amounts. Unfortunately, these bioactive lipids are prone to breakdown by enzymatic hydrolysis, which reduce or potentially reverse their positive effect. Indeed, targeting the inhibition of these catabolic routes, mediated by enzymes like fatty acid amide hydrolases (FAAH) and soluble epoxide hydrolases (sEH), has become a relevant therapeutic approach (McDougle et al., 2017; Kodani et al., 2018; Wang Y. et al., 2021). Because these bioactive lipids can be absorbed from the diet and serve such vital functions, they can be regarded as “essential bioactive dietary lipids” (Catani et al., 2018; Zhang Y. et al., 2021). Due to the abundance of precursor oils found in walnuts and the protective purpose of the walnut pellicle, this tissue is a likely reservoir of these potent bioactive lipids.

After discovering multiple species of these bioactive lipids in isolated walnut pellicles through an untargeted metabolomics approach, we sought to characterize walnut-derived bioactive lipids in the pellicle-enriched waste streams of industrial walnut processing. Focusing on oxylipins and N-acylethanolamine (NAE) fatty acids, which resemble the structure and function of human-derived endocannabinoids (like anandamide), we investigated two walnut processing waste streams that result from California’s highly mechanized post-harvest processing of walnuts. Detection of these metabolites in abundant and low-value agricultural wastes would represent a potentially untapped source of valuable bioactive lipids. The use of agricultural wastes to isolate valuable bioactive molecules or create nutraceuticals falls under waste valorization, improving industry economic output and sustainability (Aschemann-Witzel and Stangherlin, 2021). Furthermore, detecting these compounds in the pellicle sheds light on the “hidden quality” of walnuts concerning human health and well-being.

## 2 Materials and methods

### 2.1 Untargeted metabolomic profiling of isolated pellicle

**Figure 1:**
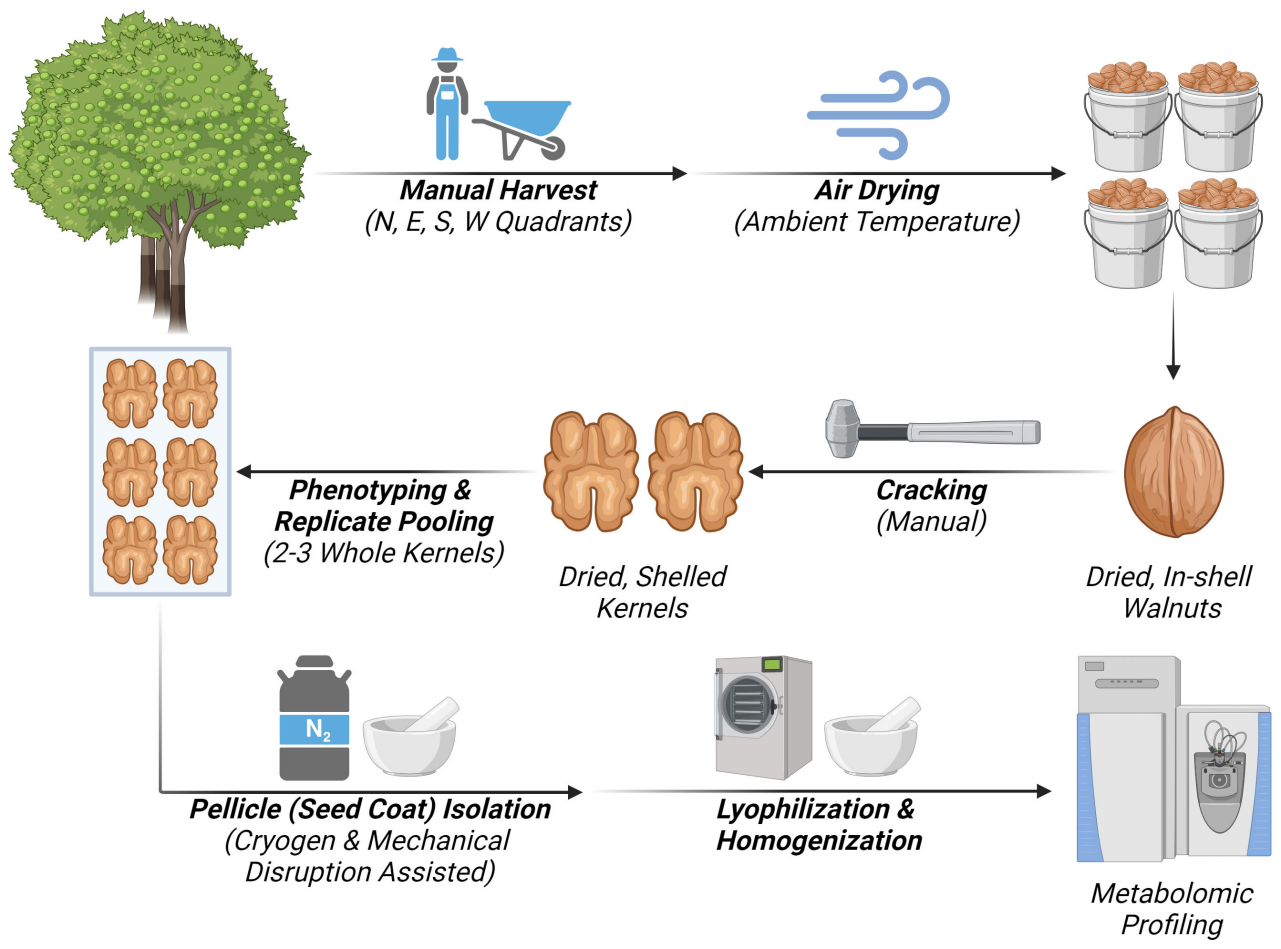
Workflow diagram representing the steps if the experimental design for untargeted metabolomic analysis of walnut seed coat. Created with BioRender.com.

The workflow diagram describing the experimental design adopted for the untargeted metabolomic profiling of walnut seed cots (pellicle) is reported in (**Figure 1**) showing the following steps: sample collection, sample storage, cracking and replicate composition, pellicle isolation and preparation, untargeted metabolomics.

#### 2.1.1 Sample collection

Physiologically mature walnut fruits were harvested from three independent trees (*Juglans regia* cv. ‘Vina’) at the University of California, Davis, courtesy of the Walnut Improvement Program. Trees one and two were physically adjacent to one another in a single block (38°32’19.6”N 121°47’45.4”W), while tree 3 was in the separate but nearby block (38°32’39.8”N 121°47’39.6”W). All three mother trees were grafted on commercial rootstocks; with tree one on Northern California Black (NCB) walnut (*Juglans hindsii*), tree two on seedling “Paradox” walnut (*J. hindsii x J. regia*), and tree three on the clonal “Paradox” RX1 (*Juglans microcarpa x J. regia*). At the time of sampling, the trees were 23, 24, and 11 years old, respectively. The block containing trees one and two were planted at lower density than the block containing tree three, and in combination with their age, trees one and two were larger with more full canopies at the time of sampling. Irrigation of these blocks began in May via micro-sprinklers, and water was applied at a near-weekly frequency with more or less water applied as needed based on local environmental and soil conditions. Based on data made available by the National Weather Service (NWS-NOAA) from a nearby monitoring station (DAVIS 2 WSW EXP FARM, CA), the annual mean temperature of the growing region was 64.2°F (Jan.-Dec.) while the growing season mean temperature was 70.7°F (Apr.-Nov.). The annual precipitation was reported as 18.57 inches^5^.

Fruit maturity was assessed by monitoring Packing Tissue Brown (PTB) and hull dehiscence. The trees were harvested by shaking once PTB was achieved, indicating full maturation of the kernels, and when greater than 50% of the canopy exhibited partial to complete dehiscence of the hull (harvest maturity stages M2 “Hull Split” and M3 “Hull Bloom”, respectively), which facilitates harvest of the in-shell nuts. Trees one and two were harvested on 9/29/2021, while tree three was harvested on 10/1/2021. Bulk samples were collected spatially around the trees in the four cardinal directions, with each quadrant bounded by the nearest ordinal directions (i.e., the “North” quadrant spanned from North-West to North-East). Nuts were collected on tarps, and immature fruit (harvest maturity stage M1 “Hull Intact”) were discarded. Fruit exhibiting hull dehiscence were manually hulled, inspected for insect or pathogen damage, and then bagged and gently air-dried at room temperature (~21°C).

#### 2.1.2 Sample storage, cracking, and replicate composition

Following a brief in-shell storage period at ambient conditions, the nuts were cracked open carefully using a small hammer, needle-nose pliers, and a metal scoopula on 12/15/2021. The two kernel halves from any given nut were retained as a pair to prevent mixing kernel halves and loss of pooled sample independence. Phenotypically uniform kernels that met the DFA of California visual grading standards 1 and 2 (“Extra Light” and “Light”, respectively) were set aside. Two to three whole kernels, comprised of two kernel halves each, were used to compose pooled replicates. Each quadrant produced one pooled replicate, yielding 12 total independent biological (pooled) replicates, with four from each mother tree. The samples were snap-frozen in liquid nitrogen and stored at -80°C to preserve their integrity for downstream processing.

#### 2.1.3 Pellicle isolation and preparation

Isolation of the pellicle from whole kernel samples was achieved through a combined method of cryogens and mechanical disruption. Whole kernels were placed into liquid nitrogen pre-chilled mortars (kept cool over a bed of dry ice) and snap-frozen with further additions of liquid nitrogen. Gentle mechanical disruption of the kernels with a pestle caused fragmentation of the frozen meat and clean separation of the pellicle from the meat. For pellicles that did not separate easily, notably around the connection point with the maternal vascular bundle (funiculus), removal was facilitated by further fragmentation and prying with forceps or a scalpel. Performed in batches, the isolation of the pellicle was completed by 6/15/2022. A workflow diagram of this process is show in **Supplementary Figure 1**, illustrated with a red-pigmented seed coat variety (*J. regia* cv. ‘Robert Livermore’) for clear visual distinction between the seed coat and nutmeat. Following isolation, the pellicle samples were lyophilized overnight in a food-grade freeze drier (Harvest Right; Model HR3000-AL). The lyophilized pellicle samples were split evenly between two 2-mL Eppendorf microcentrifuge tubes and homogenized in a laboratory mixer mill with a single 5-mm stainless steel bead for 1 minute at 30 Hz (Retsch; Model MM-400). This resulted in a fine powder ultimately used for the subsequent exploratory untargeted metabolomic analysis.

#### 2.1.4 Untargeted metabolomics

Broad metabolomic profiles of the pellicle samples were generated by the West Coast Metabolomics Center (WCMC) at UC Davis across three platforms. These included: 1) complex lipids by BEH C18-QTOF MS/MS; 2) phenolics and fatty acids by BEH C18-Q Exactive MS/MS; and 3) primary metabolites by GC-TOF MS. 10-mg of lyophilized tissue was supplied per sample and was sufficient for analysis on all three platforms.

##### 2.1.4.1 Complex lipids

###### 2.1.4.1.1 Sample preparation

Samples were extracted using the Matyash extraction procedure, including MTBE, MeOH, and H_2_O. The organic (upper) phase was dried down and submitted for resuspension and injection onto the LC, while the aqueous (bottom) phase was dried down and submitted to derivatization for GC. They were resuspended with 110 μL of a solution of 9:1 MeOH/toluene (v/v) and 50 ng/mL of 1-cyclohexyl ureido 3-dodecanoic acid (CUDA). This was then shaken (shaker, Torrey Pines Scientific Inc. Orbital Mixing Chilling/Heating Plate) for 20 seconds, sonicated for 5 minutes (Sonicor, Ultrasonic) at room temperature, and centrifuged (centrifuge Eppendorf 5425) for 2 minutes at 16100 rcf. The samples were then aliquoted into three parts: 33 μL were aliquoted into a vial with a 50 μL glass insert, for each positive and negative mode lipidomics. The last part was aliquoted into an Eppendorf tube to be used as a pool. The pieces of equipment used in this sample preparation, i.e., shaker, vortex, sonicator, and centrifuge, were adopted also for all the following experimental procedures.

###### 2.1.4.1.2 Data acquisition, LC-MS instrumentation and operating conditions

The samples were then loaded up on an Agilent 1290 Infinity LC stack (Agilent Technologies, Santa Clara, CA). The positive mode was run on a 6546 LC-QTOF (Agilent) with a scan range of m/z 120–1200 Da with an acquisition speed of 2 spectra/s. The other sample aliquot was run in negative mode, on a 6550 QTOF (Agilent) with an acquisition rate of 2 spectra/s and scan range of m/z 60–1200 Da. The mass resolution for the Agilent 6546 is 10,000 for ESI (+) and for the Agilent 6550 is 30,000 for ESI (-).

Chromatographic conditions: separations were performed using an Acquity Premier BEH C18 1.7 µm, 2.1 x 50 mm column (Waters Corp, Milford MA), in both polarities. The gradient used for both polarities is 0 min 15% (B), 0.75 min 30% (B), 0.98 min 48% (B), 4.00 min 82% (B), 4.13–4.50 min 99% (B), 4.58–5.50 min 15% (B) with a flow rate of 0.8 mL/min. ESI (+) Mobile phase A: 60:40 v/v acetonitrile:water + 10 mM ammonium formate + 0.1% formic acid Mobile phase B: 90:10 v/v isopropanol:acetonitrile + 10 mM ammonium formate + 0.1% formic acid. Chromatographic parameters for ESI (-): Mobile phase A: 60:40 v/v acetonitrile:water + 10 mM ammonium acetate Mobile phase B: 90:10 v/v isopropanol:acetonitrile + 10 mM ammonium acetate. For both operating modes, column temperature was set constant at 65°C.

###### 2.1.4.1.3 Spectral data processing and compound identification

The general workflow for data processing used MS-DIAL (Tsugawa et al., 2015), followed by a blank subtraction in Microsoft Excel and a cleanup of data using MS-FLO (DeFelice et al., 2017). The first step was to convert files using the Abf Converter. Default parameters were used to process MS-Dial data, except for minimum peak height and width, adjusted for the instrument where the samples ran. Once the results have been exported from MS-DIAL, a blank reduction was made based on the max peak height relative to the blank average height, the average of all non-zero peak heights for samples, and if the feature was found in at least one sample. Next, using MS-FLO, potential duplicates, and isotopes were checked and deleted if confirmed. Then, MS/MS spectra were checked before combining adducts. Peaks were annotated in manual comparison of MS/MS spectra and accurate masses of the precursor ion to spectra given in the Fiehn laboratory’s LipidBlast spectral library (Kind et al., 2013). Additional peaks were found by manually curating sample chromatograms on a scan-by-scan basis. MassHunter Quant software (Agilent) was then used to verify peak candidates based on peak shape, peak height reproducibility, and retention time reproducibility in replicate samples. Valid and reproducible peaks were analyzed by targeted MS/MS to increase overall peak annotations in both positive and negative modes.

##### 2.1.4.2 Phenols and other fatty acid derivatives

The samples were injected onto a Waters Acquity Premier BEH C18 1.7 µm, 2.1 x 50 mm column (Waters). The gradient used was 0 min 1% (B), 0.50 min 1% (B), 7.50 min 99% (B), 9.00 min 99% (B), 9.20 min 1% (B), 10.00 min 1% (B), with a flow rate of 0.6 mL/min. Mobile phase A was 100% LC/MS grade water + 0.1% Formic Acid, and mobile phase B was 100% ACN + 0.1% Formic Acid. Injection volume varies by study and ranges between 0.1 μL and 5 μL. A Vanquish UHPLC system (ThermoFisher Scientific, Waltham MA) was used. A Thermo Q-Exactive HF Orbitrap MS instrument (ThermoFisher) was used in both positive and negative ESI modes to acquire LC-MS/MS data with the following parameters: mass range 80−1200 m/z; full scan MS1 mass resolving power 60,000, data-dependent MSMS (dd-MSMS) 2 scans per cycle (4 scans per cycle for pooled MSMS injections), normalized collision energy at 20%, 30%, and 40%, dd-MSMS mass resolving power 15,000.

###### 2.1.4.2.1 Sample preparation

Samples were extracted using 1 mL of 80:20 MeOH/H_2_O (v/v). Samples were vortexed and centrifuged. 450 μL of the supernatant was dried for analysis. Dried samples were resuspended with 100 μL of a solution 75:25 H_2_O/ACN containing internal standards (CUDA, D3-L-Carnitine, Val-Tyr-Val, D4-Daidzein, D9-Reserpine, and D5-Hippuric Acid). Samples were then vortexed for 10 seconds, sonicated for 5 minutes at room temperature, and then centrifuged for 2 minutes at 16,000 rcf. 60 μL supernatant from each sample was transferred into an LC-MS vial containing a glass micro insert. 30 μL supernatant from each sample was then transferred into an Eppendorf tube and vortexed for use as a pool.

###### 2.1.4.2.2 Data acquisition, LC-MS instrumentation and operating conditions

The samples were then injected onto a Waters Acquity Premier BEH C18 1.7 µm, 2.1 x 50 mm column (Waters). The gradient used was 0 min 1% (B), 0.50 min 1% (B), 7.50 min 99% (B), 9.00 min 99% (B), 9.20 min 1% (B), 10.00 min 1% (B), with a flow rate of 0.6 mL/min. Mobile phase A was 100% LC/MS grade water + 0.1% Formic Acid, and mobile phase B was 100% ACN + 0.1% Formic Acid. Injection volume varies by study and ranges between 0.1 μL and 5 μL. Vanquish UHPLC system (ThermoFisher Scientific) was used. A Thermo Q-Exactive HF Orbitrap MS instrument (ThermoFisher Scientific) was used in both positive and negative ESI modes to acquire LC-MS/MS data with the following parameters: mass range 80−1200 m/z; full scan MS1 mass resolving power 60,000, data-dependent MSMS (dd-MSMS) 2 scans per cycle (4 scans per cycle for pooled MSMS injections), normalized collision energy at 20%, 30%, and 40%, dd-MSMS mass resolving power 15,000.

##### 2.1.4.3 Primary metabolites

###### 2.1.4.3.1 Sample preparation

Samples were extracted using the Matyash extraction procedure, which includes MTBE, MeOH, and H_2_O. The organic (upper) phase was dried down and submitted for resuspension and injection onto the LC, while the aqueous (bottom) phase was dried down and submitted to derivatization for GC. They are shaken at 30°C for 1.5 hours. Then 91 μL of MSTFA + FAMEs were added to each sample and shaken at 37°C for 0.5 hours to finish derivatization. Samples are then vialed, capped, and injected into the instrument.

###### 2.1.4.3.2 Data acquisition, GC-MS instrumentation and operating conditions

A 7890A GC (Agilent) coupled with a Pegasus TOF MS (LECO Corp, St. Joseph, MI) was used. 0.5 μL of derivatized sample was injected using a splitless method onto a RESTEK RTX-5SIL MS column with an Intergra-Guard (Restek Corp, Centre County, PA) at 275°C and a 1 mL/min helium flow. The GC oven was set to hold at 50°C for 1 minute, then ramp to 20°C/minute to 330°C, then hold for 5 minutes. The transfer line was set to 280°C while the EI ion source is set to 250°C. The mass spectrometer parameters collected data from 85m/z to 500m/z at an acquisition rate of 17 spectra/sec.

###### 2.1.4.3.3 Spectral data processing and compound identification

Raw data files were preprocessed directly after data acquisition and stored as ChromaTOF-specific *.peg files, generic *.txt result files, and generic ANDI MS *.cdf files. ChromaTOF vs. 2.32 (LECO) was used for data preprocessing without smoothing, 3 s peak width, baseline subtraction just above the noise level, and automatic mass spectral deconvolution and peak detection at signal/noise levels of 5:1 throughout the chromatogram. Apex masses were reported for use in the BinBase algorithm. Result *.txt files were exported to a data server with absolute spectra intensities and further processed by a filtering algorithm implemented in the metabolomics BinBase database. The BinBase algorithm (rtx5) used the settings: validity of chromatogram (<10 peaks with intensity >10^7 counts s-1), unbiased retention index marker detection (MS similarity>800, validity of intensity range for high m/z marker ions), retention index calculation by 5th order polynomial regression. Spectra were cut to 5% base peak abundance and matched to database entries from most to least abundant spectra using the following matching filters: retention index window ±2,000 units (equivalent to about ±2 s retention time), validation of unique ions and apex masses (unique ion must be included in apexingmasses and present at >3% of base peak abundance), mass spectrum similarity must fit criteria dependent on peak purity and signal/noise ratios and a final isomer filter. Failed spectra were automatically entered as new database entries if s/n >25, purity <1.0, and presence in the biological study design class was >80%. All thresholds reflect settings for ChromaTOF v. 2.32. Quantification was reported as peak height using the unique ion as the default unless a different quantification ion was manually set in the BinBase administration software BinView. A quantification report table was produced for all database entries that are positively detected in more than 10% of the samples of a study design class (as defined in the miniX database) for unidentified metabolites. A subsequent post-processing module was employed to replace missing values from the *.cdf files automatically. Replaced values were labeled as ‘low confidence’ by color coding. For each metabolite, the number of high-confidence peak detections and the ratio of the average height of replaced values to high-confidence peak detections were recorded. These ratios and numbers were used for the manual curation of automatic report data sets to data sets released for submission index.

##### 2.1.4.4 Metabolite annotation

The analytical approaches described in section 1.4 enabled the detection of 1595 features, 5274 features, and 534 features for the three platforms. However, the number of known, identified metabolites was 216 features (60 in negative mode and 156 in positive mode), 221 (88 in negative mode and 133 in positive mode), and 156 features in each dataset from the three platforms, respectively. Each feature was investigated and manually annotated from the provided lists of identified metabolites. Querying publicly accessible information from KEGG (Kyoto Encyclopedia of Genes and Genomes), NIH-PubChem (National Institute of Health), and HMDB (Human Metabolome Database) resources with international chemical identifier (InChI) keys facilitated the acquisition of database identifiers, biochemical pathway information, and molecular structure information for each metabolite. Each metabolite was annotated with KEGG and PubChem identifiers, a “superpathway” and related “subpathway(s)” based on their known involvement in biological pathways, and biochemical classifications and related subclassifications based on molecular structure and functional groups. The complete set of curated annotations can be found in **Supplementary Table 4**.

### 2.2 Targeted metabolomics of pellicle-enriched industry waste by-products

#### 2.2.1 Waste by-products acquisition

**Figure 2:**
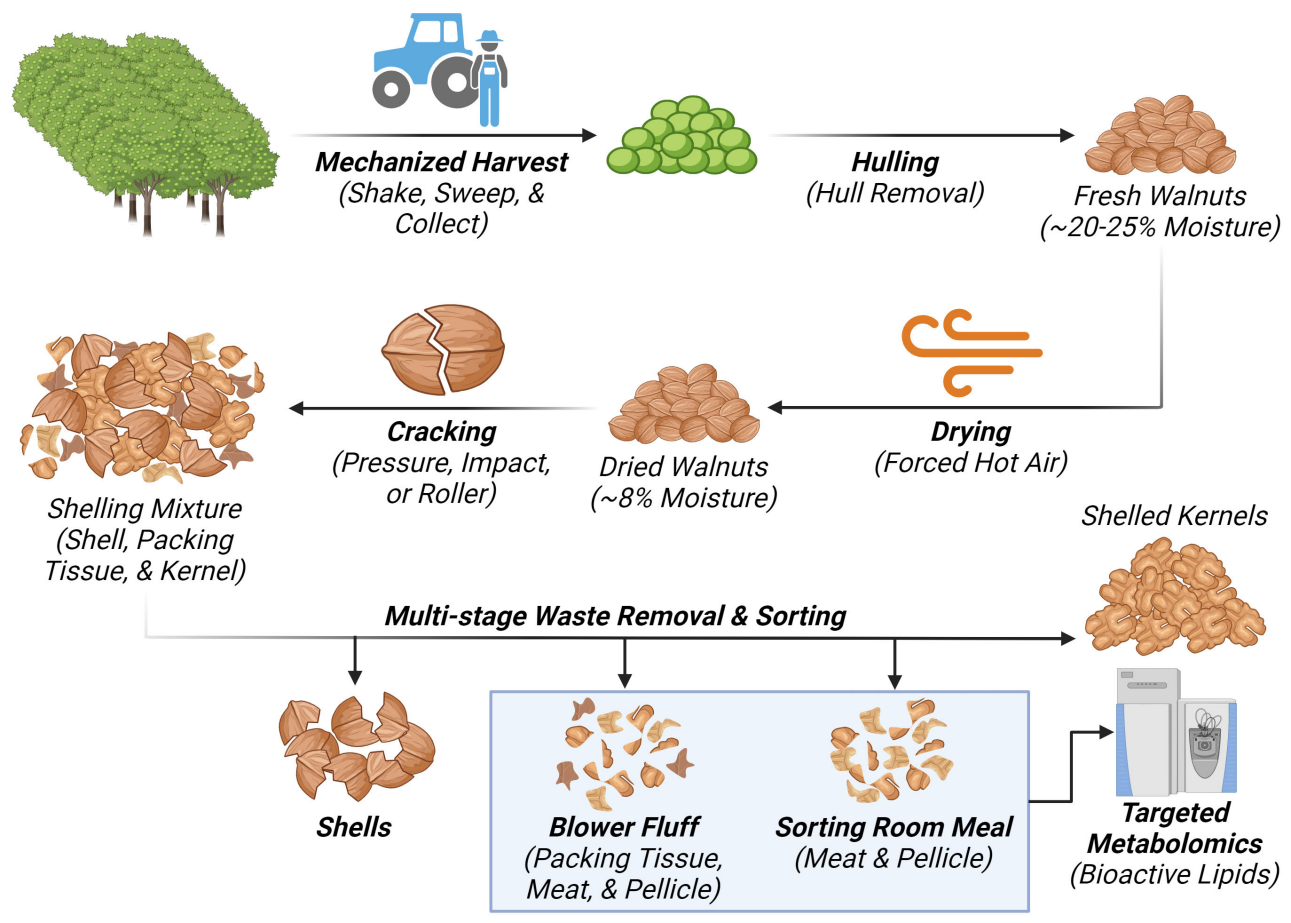
Diagram representing the walnut processing waste stream generating “Blower Fluff (BF)” and “Sorting room Meal (SRM)” samples respectively. Created with BioRender.com

Carriere Family Farms kindly provided pellicle-enriched waste by-products from the mechanized industrial processing of walnuts. These by-products consisted of two different waste streams: “blower fluff” (BF) and “sorting room meal” (SRM), which come from earlier and later processing stages, respectively. The BF waste stream is generated at the end of the shelling process and is comprised of small shell pieces, dried packing tissue, nutmeat pieces, and pellicle fragments (**Figure 2**). By weight, these components made up approximately 59%, 30%, and 11%, respectively. The SRM waste stream comes from the subsequent sorting of shelled kernels. It is composed almost exclusively of nutmeat pieces and pellicle fragments, with by-weight percentages of approximately 67% and 33%, respectively. Though both waste classes are composed of other walnut tissues besides the pellicle, these are two of the most pellicle-enriched wastes from the mechanized processing of walnuts in California. The bulk samples, sealed in air-tight plastic bags, were immediately stored at –80°C to maintain their integrity and were submitted for metabolomic analysis within one month of acquisition.

#### 2.2.2 Waste by-product sample preparation

Three (technical) replicates were taken from each bulk sample for each waste stream and used to fuel a targeted metabolomics analysis. This 127-member panel included oxylipins (92), N-acylethanolamines (NAEs) (30), and polyunsaturated fatty acids (PUFAs) (5). No further processing of the samples was performed before LC-MS analysis as the type of wastes selected for investigation were already commercially dried and in the form of a coarse powder.

#### 2.2.3 Targeted metabolomic panel

##### 2.2.3.1 Sample preparation and lipid extraction

Complex lipids were extracted with 8:10:11 isopropanol/cyclohexane/ammonium acetate, followed by alkaline hydrolysis and isolation by solid phase extraction using modifications of previously reported methods (Newman et al., 2014). Briefly, ~50 mg ground waste byproduct was mixed with 5µL 0.2 mg/mL butylated hydroxytoluene/EDTA and homogenized in 410 µL isopropanol on a vertical ball mill (GenoGrinder 2010, SPEX SamplePrep, Metuchen, NJ). Samples were mixed with 520 µL cyclohexane by vortexing for 3 min. The homogenate was then transferred to a 2-mL polypropylene 96-well plate. The homogenate was mixed with 570 µL of 0.1 M ammonium acetate, vortexed for 3 min, and centrifuged for 5 min at 2.3 g and 4°C. The organic phase was transferred to a new plate, and the aqueous phase was re-extracted with 520 µL cyclohexane. The combined organic phases were evaporated under vacuum, and the residue was dissolved in 100 µL 1:1 methanol/toluene (v/v).

These total lipid extracts were then enriched with a suite of deuterated oxylipin free acids, incubated with 100 µL of 0.5 M sodium methoxide for 50 min at 50°C, mixed with 100 µL water, and returned to 50°C for 50 min. Samples were neutralized with 10 µL 20% glacial acetic acid and then diluted with 1 mL 0.1% acetic acid/5% methanol, and oxylipins were trapped on a 10 mg Oasis HLB solid phase extraction column (Waters). After washing with 2 mL 0.1% acetic acid/30% MeOH, analytes were eluted in 250 µL methanol with 1% acetic acid followed by 1 mL ethyl acetate and collected into plate wells containing 10 µL pf 20% glycerol in methanol. Solvents were removed by vacuum evaporation, and residues were reconstituted in 125 µL of CUDA and 1-phenyl ureido 3-hexanoic acid (PUHA) (Cayman Chemical; Ann Arbor, MI) at 100 nM in 1:1 methanol:acetonitrile. Samples were chilled at -20°C for 15 min, filtered with 0.2 µm PVDF 96-well plates (Agilent), and then stored at -20°C until analysis within 48 h.

##### 2.2.3.2 UPLC-MS/MS acquisition and operating conditions

Residues in extracts were separated on a 2.1 mm x 150 mm, 1.7 µm Acquity BEH C18 column (Waters, Milford, MA) and detected by electrospray ionization with scheduled multiple reaction monitoring on 6500 QTRAP tandem mass spectrometer (Sciex; Redwood City, CA) as previously described (Pedersen et al., 2021). A binary gradient elution program was adopted based on water with 0.1% Acetic Acid (solvent A) and 90% Acetonitrile/10% isopropanol (solvent B). The flow rate was always set at 0.5 mL/min and the column temperature at 60°C. Analytes were quantified using isotope dilution and internal standard ratio-response methodologies against a minimum 7 pt calibration curve bracketing reported concentrations. F2-isoprostanes (F2isoPs) were detected as a complex cluster of analytes with the same mass transition as and surrounding PGF2a. F2-IsoP concentrations were estimated using the response of prostaglandin F2a (353.3 > 193.2 m/z) as previously described (Keenan et al., 2012). Refer to **Supplementary Table 6** for more details.

##### 2.2.3.3 Data quality and reporting

Analytical results generally met quality control criteria concerning surrogate recoveries and replicate precision. Method performance was assessed through the routine analysis of blanks using analytical methods corrected for the performance of analytical surrogates. While apparent surrogate recoveries were ~20–50%, lower than generally observed for animal tissues, they were deemed acceptable. Experimental replicates appeared reasonable. Results are expressed in nmol/g tissue (i.e., µM) except for a subset of analytes, including polyunsaturated fatty acids and six alpha-linolenic acid-derived oxylipins for which commercially available standards were not available: These analytes (denoted as screens) are reported as relative abundance across all measured samples (i.e., the sum of each metabolite across all samples is set to 100%). Out of 127 analytes panel, 45% were present in the samples and fulfilled all QC requirements, whereas 30% of the analytes were not detected. 9-HODE and 13-KODE were above the highest calibration standard, and results were estimated by linear extrapolation of the calibration curve and should, therefore, represent a minimum concentration and may be associated with less accuracy. Refer to **Supplementary Table 6** for more details.

### 2.3 Bioinformatics and data availability

All analysis and visualizations were performed in GraphPad PRISM v10.1.1 (GraphPad Software, La Jolla, CA).

The curated annotations were used to explore the composition of major biochemical classes and subclasses within the untargeted metabolome of the walnut pellicle. Summarization of the data included counting feature membership in various biochemical classes, subclasses, and structurally related features within a class or subclass. Each biochemical class or subclass was represented as a relative percentage over the total number of the identified features in the dataset or a specific class or subclass.

For each dataset (representing independent analytical platforms), the relative intensity of each metabolite was expressed as percentages of the total intensity for their respective platform. The total un-normalized intensity was calculated using all of the data per platform, including unidentified features (**Supplementary Table 7**). The total intensity for each sample was determined, and then the average of these total intensities (n = 12) was calculated as the global average intensity. The average intensity across all samples (n = 12) was calculated for every metabolite and then divided by the global average intensity. These results were multiplied by 100 to express them as percentages. To understand the relative intensity of multiple metabolites to the other members within their family, the total dataset was filtered down to just members of the oxylipins, NAEs, and DA-FAs. The same calculations described above were performed to determine the percentage intensity of each query metabolite among the narrower groups.

Concerning the targeted panel, Welch’s t-test (p < 0.05) was used to determine statistical differences in the bioactive lipid concentrations between the two waste byproducts (**Supplementary Table 5**). No corrections for multiple comparisons were employed due to the small number of technical replicates for each class.

The raw data of the untargeted metabolomic profile are given as peak heights for the quantification ion (mz value) at the specific retention time (rt value) (**Supplementary Tables 1**–**3**). Concentrations of the detected oxylipins and endocannabinoids results of the targeted investigation are reported in **Supplementary Table 5**.

## 3 Results

### 3.1 Global metabolic profile of the walnut pellicle

Exploration of the global metabolic profile of the walnut pellicle was enabled by a triple-platform analytical pipeline, as described in the Materials and Methods section. A total of 7403 features were detected, with 1595 features identified with *Platform 1* (“Complex Lipid Analysis”), 5274 with *Platform 2* (“Polyphenol and Fatty Acid Analysis”), and 534 with *Platform 3* (“GC-MS Analysis for primary Metabolism”). From this total number of detected features, 565 unique metabolites (216, 221, and 156 from *Platforms 1, 2*, and *3*) were successfully identified.

**Figure 3:**
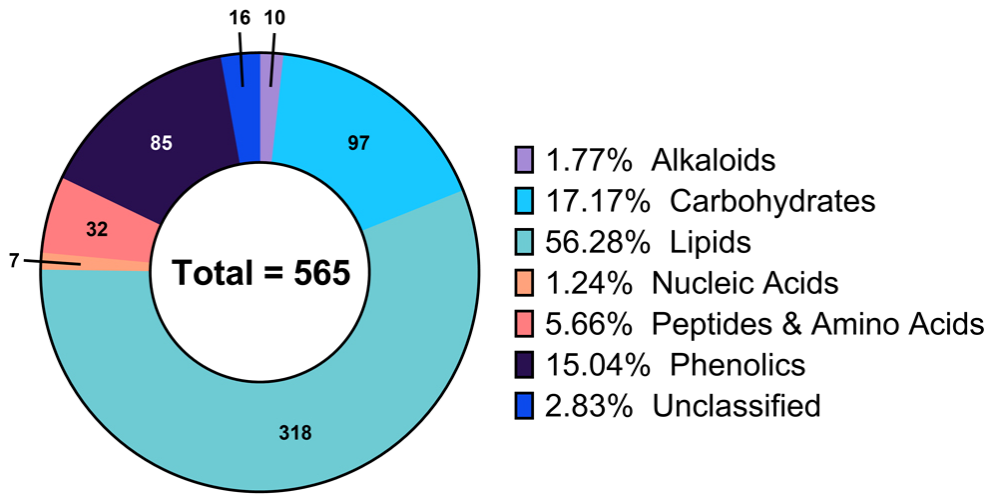
Composition of the global metabolic profile of walnut pellicle expressed as percentage of the total detected and identified metabolites.

Annotations for each metabolite, including biochemical pathway membership and classification by molecular structure, were used to summarize the metabolomic profile of the pellicle. As shown in **Figure 3**, 56.28% (318) of the identified metabolites were classified as lipids, followed by carbohydrates at 17.17% (97), phenolics at 15.04% (85), peptides and amino acids at 5.66% (32), alkaloids at 1.77% (10) and lastly nucleic acids at 1.24% (7). Seed coat metabolomic studies have generally been interested in the phenolics fraction due to their role in barrier function, contributing antioxidant and antimicrobial properties. This has also largely been the case for walnuts, with pellicle (and shell and hull) metabolomics focused primarily on the diversity of phenolics contributing to antioxidant capacity. To our knowledge, this investigation is the first deep metabolomic profiling of the walnut pellicle, revealing a vast repertoire of diverse lipids and lipid-derived compounds.

### 3.2 General overview of major lipid classes in the walnut pellicle

**Figure 4:**
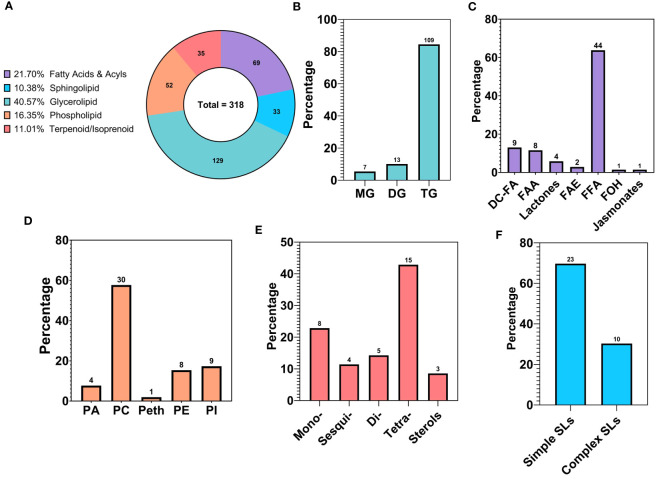
Compositional analysis of the lipid profile of walnut pellicle: **(A)** Lipid classes expressed as relative percentage of the total detected and identified metabolites. **(B)** Subclasses of Glycerolipids (GLs) expressed as % of the total features belonging to the GL class. **(C)** Subclasses of Fatty Acids and Acyls expressed as % of the total features belonging to the Fatty Acids and Acyl class. **(D)** Subclasses of Phospholipids (PLs) expressed as % of the total features belonging to the PL class. **(E)** Subclasses of Terpenes expressed as % of the total features belonging to the Terpenes class. **(F)** Subclasses of Sphingolipids (SLs) expressed as % of the total features belonging to the SL class. MG, Monoacylglycerol; DG, Diacylglycerol; TG, Triacylglycerol; DC-FA, Dicarboxylic Fatty Acid; FAA, Fatty Acid Amide; FAE, Fatty Acid Esters; FFA, Free Fatty Acid; FOH, Fatty alcohol.

The lipid fraction accounted for a total of 318 unique metabolites, which were identified as lipid molecules using their biochemical class annotations. The footprint of each major lipid class, represented as metabolite counts and relative percentage of the overall lipids, can be seen in **Figure 4A**. These major classes encompass glycerolipids, fatty acids (and fatty acyls), phospholipids, terpenoids, and sphingolipids.

The glycerolipid (GL) class was the most abundant of the lipid classes, accounting for 40.57% (129) of the total lipid features. This class includes triacylglycerols (TG), diacylglycerols (DG), and monoacylglycerols (MAG), which were represented at 84.50% (109), 10.08% (13), and 5.43% (7) of the total GL class, respectively (**Figure 4B**). Within the DGs, multiple species of glycolipids were detected and comprised 3.1% and 1.26% of the GLs and total lipid features, respectively. Of particular note are glycerolipids containing a glucuronic acid moiety, specifically two diacylglycerol glucuronide molecules named DGGA 34:2 (16:0/18:2) and DGGA 36:3 (18:1/18:2). This peculiar subclass of GLs has, to the best of our knowledge, never been reported in the walnut pellicle.

**Figure 5:**
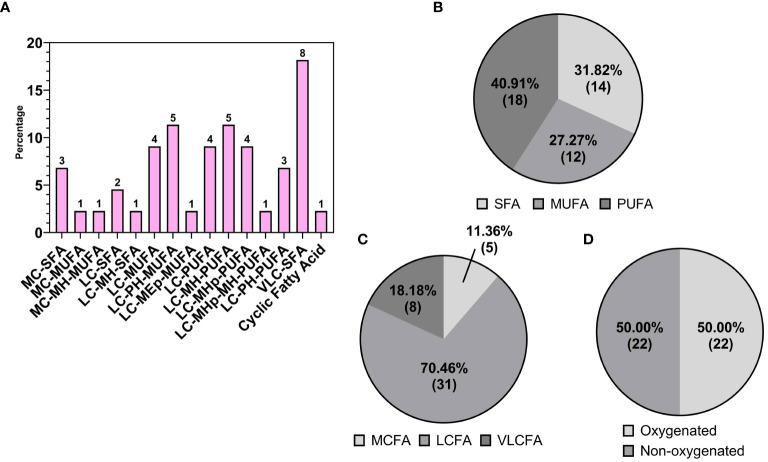
**(A)** Detailed subgrouping of Free Fatty Acids (FFAs): medium chain saturated fatty acid (MC-SFA); medium chain monounsaturated fatty acids (MC-MUFA); medium chain monohydroxy monounsaturated fatty acids (MC-MH-MUFA); long chain saturated fatty acids (LC-SFA); long chain monohydroxy saturated fatty acids (LC-MH-SFA); long chain monounsaturated fatty acids (LC-MUFA); long chain polyhydroxy monounsaturated fatty acids (LC-PH-MUFA); long chain monoepoxy monounsaturated fatty acids (LC-MEp-MUFA); long chain polyunsaturated fatty acids (LC-PUFA); long chain monohydroxy polyunsaturated fatty acids (LC-MH-PUFA); long chain monohydroperoxy polyunsaturated fatty acids (LC-MHp-PUFA); long chain monohydroperoxy monohydroxy polyunsaturated fatty acids (LC-MHp-MH-PUFA);long chain polyhydroxy polyunsaturated fatty acids (LC-PH-PUFA); very long chain fatty acids (VLC-FA). Subspecies of FFAs on the base of **(B)** saturation: saturated (SFA), monosaturated (MFA), polyunsaturated (PUFA); **(C)** length: short chain (SCFA), medium chain (MCFA), long chain (LCFA); **(D)** oxygenation of the FFA carbon chains.

Fatty acids, belonging to the “Fatty Acids and Acyls” class, were the second most frequently identified group in the walnut seed coat, representing 21.70% (69) of the total lipid fraction expressed in counts (**Figure 4A**). A rich and highly diverse array of free fatty acids (FFAs) were identified, accounting for 63.77% (44) of the total fatty acids (**Figure 4C**). FFAs diversity was explored by grouping metabolites according to their structural characteristics, including carbon chain length, chemical modification of the carbon chain, and degree of unsaturation. The majority of FFAs, up to 70.46% (31) of the total fatty acid counts, were long-chain fatty acids, with chain lengths between 13–21 carbon atoms, followed by medium chain 11.36% (5) with chain lengths of 6–12 carbon atoms, and very long chain fatty acids 18.18% (8) with chain lengths ≥ 22 carbon atoms (**Figure 5C**). The distribution of unsaturation degree of the carbon chain in the FFAs population is shown in **Figure 5B**; 40.91% (18), 27.27% (12), and 31.82% (14) accounted for by polyunsaturated FFAs (PUFAs), monounsaturated FFAs (MUFAs), and saturated FFAs (SFAs) respectively, including both oxygenated and non-oxygenated FFA (**Figure 5B**). Notably, half of the FFA, 50% (22), possessed oxygenated carbon chains. These included mono- and poly-hydroxy FFAs and mono-hydroxyperoxy and epoxy FFAs (**Figure 5D**). Typical non-oxygenated long-chain monosaturated fatty acids (LC-MUFAs) and long-chain polyunsaturated fatty acids (LC-PUFAs), which include oleic, linoleic, and linolenic acids, accounted each for just 9% (4) of the total FFAs; similarly, the low count was also found for simple long-chain saturated fatty acids (LC-SFAs) at 4.55% (2) of the total FFAs identified (**Figure 5A**). In contrast, very long-chain fatty acids (VLC-FAs) counted up to the 18.18% (8) of the total FFAs.

This larger class of fatty acids also encompassed more unusual fatty acids belonging to the classes of dicarboxylic fatty acids (DC-FAs) at 13.04% (9), fatty acid amides (FAAs) at 11.59% (8), lactones at 5.79% (4), fatty acid esters (FAEs) at 2.90% (2), and lastly fatty alcohols (FOHs) and jasmonates at 1.45% (1) (**Figure 4C**). To our knowledge, this is the first time these subclasses of fatty acids were detected in walnut seed coat, in particularly the DC-FAs and lactones, along with a substantial percentage of oxylipins, in walnut-derived tissue.

Phospholipids (PLs), terpenoids/isoprenoids, and sphingolipids (SLs) represented 16.35% (52), 11.01% (35), and 10.38% (33) of the total number of lipid features, respectively (**Figure 4A**). Concerning the PLs, phosphatidylcholines (PCs) were the most represented subclass, with 57.69% (30) of the total PL population. PCs were followed by phosphatidylinositols (PIs) at 17.31% (9), phosphatidylethanolamines (PEs) at 15.38% (8), phosphatidic acids (PAs) at 7.69% (4), and finally phosphatidylethanols (Peths) at 1.92% (1) (**Figure 4D**). A noteworthy subclass of PLs, the lysophospholipids (lyso-PLs), were also identified in this study primarily contributed by the PCs class; in total five Lyso-PCs were identified counting for the 16.67% of the total PC features and 9.67% of the total PL population (**Supplementary Table 4**). Concerning the class of terpenoids/isoprenoids, membership was mainly represented by tetraterpenoids which reached up to 42.86% (15), followed by monoterpenoids at 22.86% (8), diterpenoids at 14.29% (5), sesquiterpenoids at 11.43% (4), and sterols at 8.57% (3) (**Figure 4E**). Lastly, the SL class was accounted for mainly by simple sphingolipids (sphingoid bases and ceramides) comprising 69.70% (23) of the total SL population. In comparison, complex sphingolipids (sphingomyelins and glycosphingolipids) accounted for the remaining 30.30% (10) of the total SL features (**Figure 4F**).

As a general conclusion, the qualitative compositional lipid profile of the walnut pellicle followed the order GL > FA > PL > Terpenoids/Isoprenoids > SL. GL and FA were the lipid classes most abundant in feature counts, with the TG and FFA subclasses contributing the highest counts of molecular species, respectively. Above all, the fatty acid profile of the walnut seed coat showed more insightful peculiarities compared to the commonly detected and extensively characterized fatty acids, like linoleic and linolenic acids, in the whole walnut kernel.

### 3.3 Characterization of fatty acid species in the walnut pellicle

**Figure 6:**
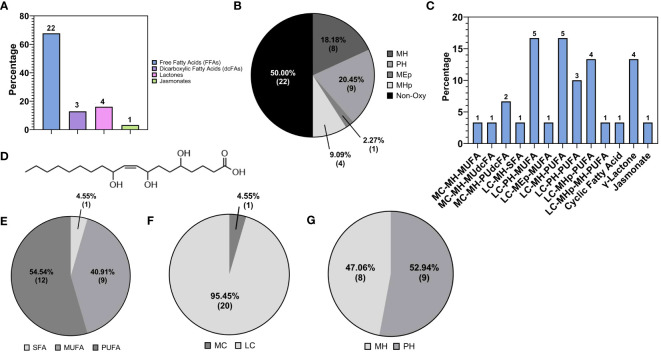
**(A)** Subclasses of oxylipins. **(B)** Subspecies of free Fatty Acids (FFAs) based on type of oxygenation, monohydroxy (MH), polyhydroxy (PH), mono-epoxy (MEp), monohydroperoxy (MHp), non-oxygenated (Non oxy). **(C)** Detailed subgrouping of oxylipins: medium chain monohydroxy monounsaturated fatty acids (MC-MH-MUFA); medium chain monohydroxy monounsaturated dicarboxylic fatty acids (MC-MH-MU-dc-FA); medium chain monohydroxy polyunsaturated dicarboxylic fatty acids (MC-MH-PU-dc-FA); long chain monohydroxy saturated fatty acids (LC-MH-SFA); long chain polyhydroxy monounsaturated fatty acids (LC-PH-MUFA); long chain monoepoxy monounsaturated fatty acids (LC-MEp-MUFA); long chain polyhydroxy polyunsaturated fatty acids (LC-PH-PUFA); long chain monohydroxy polyunsaturated fatty acids (LC-MH-PUFA); long chain monohydroperoxy polyunsaturated fatty acids (LC-MHp-PUFA); long chain monohydroperoxy monohydroxy polyunsaturated fatty acids (LC-MHp-PUFA) **(D)** Molecular structure of the most abundant FFA’s oxylipin reported in ‘**Table 2**’, namely (9Z)-5,8,11-Trihydroxyoctadec-9-enoic acid, a trihydroxy fatty acid derivative of C18:1. Subgroups of FFA’s oxylipin class on the base of **(E)** saturation: saturated (SFA), monosaturated (MFA), polyunsaturated (PUFA); **(F)** length: short chain (SCFA), medium chain (MCFA), long chain (LCFA); **(G)** type of hydroxylation of the hydroxylated free fatty acids carbon chains.

The analytical pipeline unveiled the detection of oxygenated fatty acids, i.e., oxylipins, up to 43.47% (30) of the total fatty acids. As shown in **Figure 6A**, most oxylipins were detected in the FFA class, followed by the lactones, DC-FAs and jasmonates classes. Compositionally, oxygenated FFAs accounted for 50% of the total FFA features (**Figure 5D**). As mentioned in the previous section, the types of oxygenation forms included mono-, di-, poly-hydroxylation, mono-hydroperoxidation, and mono-epoxidation (**Figure 6B**). To summarize, the majority of detected FFA oxylipins had long carbon chains, one or more unsaturation’s, and one or more oxygenations (**Figures 6E–G**). Looking at the combinations of these structural hallmarks, the majority of identified FFA oxylipins were long-chain polyhydroxy monounsaturated fatty acids (LC-PH-MUFA), followed by long-chain monohydroxy polyunsaturated fatty acids (LC-MH-PUFA), and long-chain mono-hydroperoxy polyunsaturated fatty acids (LC-MHp-PUFA). The list of metabolites belonging to the LC-PH-MUFA subclass is reported in **Table 1**, along with each metabolite’s relative abundance (%) to the global normalized intensities and molecular structures (**Figure 6D**).

**Table 1:** Major oxylipin derivatives of Free fatty acids (FFAs) with their names, m/z, type of ionization and relative abundance (%) calculated as explained in section 2.3 of Material and Methods.

Metabolite Name	m/z	Ionization	Relative Abundance %
9-Hydroperoxy-10E,12Z,15Z-octadecatrienoic acid	293.2110	[M+H-H_2_O] +	0.33
13S-Hydroxy-9Z,11E,15Z-octadecatrienoic acid	277.2162	[M+H-H_2_O] +	0.39
(Z)-9,12,13-Trihydroxyoctadec-15-enoic acid	277.2158	[M+H-3H_2_O] +	0.40
(E)-3,10-Dihydroxy-4,9-dimethyldodec-6-enedioic acid	289.1644	[M+H] +	1.67
(9E,11Z)-8-Hydroxyoctadeca-9,11-dienoic acid	279.2317	[M+H-H_2_O] +	0.55
9S-Hydroxy-10E,12Z,15Z-octadecatrienoic acid	293.2123	[M-H]-	0.36
9S-Hydroperoxy-10E,12Z-octadecadienoic acid	293.2124	[M-H-H_2_O]-	1.31
9,10-Dihydroxy-12Z-octadecenoic acid	313.2386	[M-H]-	0.50
(9Z)-5,8,11-Trihydroxyoctadec-9-enoic acid	329.2331	[M-H]-	57.25

**Figure 7:**
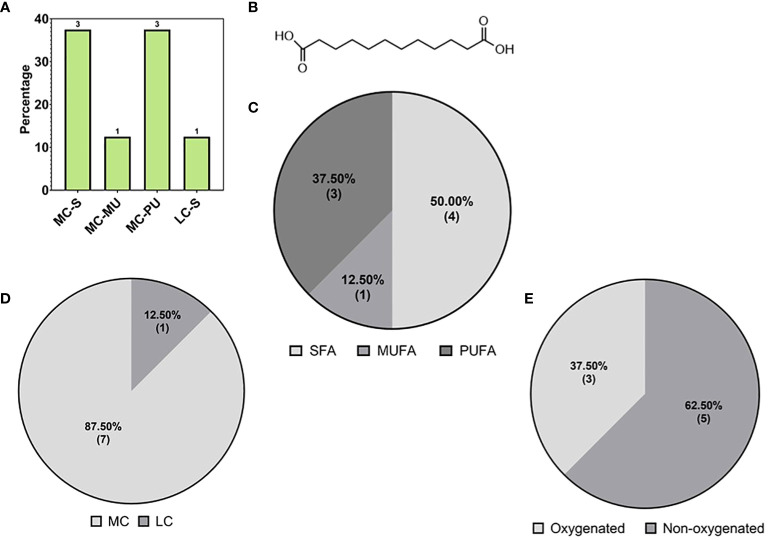
**(A)** Subspecies of dicarboxylic fatty acids (DC-FAs) on the base of carbon chain length and saturation: medium chain-saturated (MC-S), medium chain -unsaturated (MC-MU), medium chain polyunsaturated (MC-PU), long chain saturated (LC-S). **(B)** Molecular structure of the most abundant medium DC-FA reported in ‘**Table 2**’, namely Dodecanedioic acid. Subspecies of DC-FAs class on the base of **(C)** saturation: short chain (SCFA), medium chain (MCFA), long chain (LCFA); **(D)** length: short chain (SCFA), medium chain (MCFA), long chain (LCFA); **(E)** oxygenation of the DC-FA carbon chains.

The second major subclass of fatty acids detected in this study were the DC-FAs (**Figure 4C**), which were also structurally classified based on chain length, degree of saturation, and chemical modification. DC-FAs were detected mainly as medium chains with similar frequencies of mono- and poly-unsaturation’s (**Figures 7A, C, D**). While the majority were non-oxygenated, several oxylipins of DC-FAs were detected, making up 37% of the total DC-FAs features (**Figure 7E**). These results are seen in **Table 2**, which reports a list of the identified DC-FAs in the walnut pellicle along with their relative abundance (%) and molecular structures (**Figure 7B**). Interestingly, methyl esters of DC-FAs were also identified and classified under the fatty acid ester (FAE) class.

**Table 2:** Major dicarboxylic fatty acids (DC-FAs) with their names, m/z, type of ionization and relative abundance (%) calculated as explained in section 2.3 of Material and Methods.

Metabolite Name	m/z	Ionization	Relative Abundance %
Azelaic acid	317	EI	29.30
Adipic acid	111	EI	70.70
Dodecanedioic acid	195.1379	[M+H-2H_2_O] +	44.08
(2E,5E)-3,5,7-Trimethylocta-2,5-dienedioic acid	149.096	[M+H-CH_4_O_3_] +	9.52
1-O-((2E,4E)-9-Carboxy-8-hydroxy-2,7-dimethylnona-2,4-dienoyl)-. beta. -D-glucopyranose	241.1082	[M-H-C_6_H_10_O_5_]-	3.60
(4E)-8-(.beta. -D-Glucopyranosyloxy)-2,7-dimethyldec-4-enedioic acid	405.1768	[M-H]-	7.47

DC-FAs were identified in Platform 2 by LC-ESI-MS analysis, except Azelaic Acid and Adipic acid were identified in the Platform 3, by GC-MS where EI stands for electron impact.

**Figure 8:**
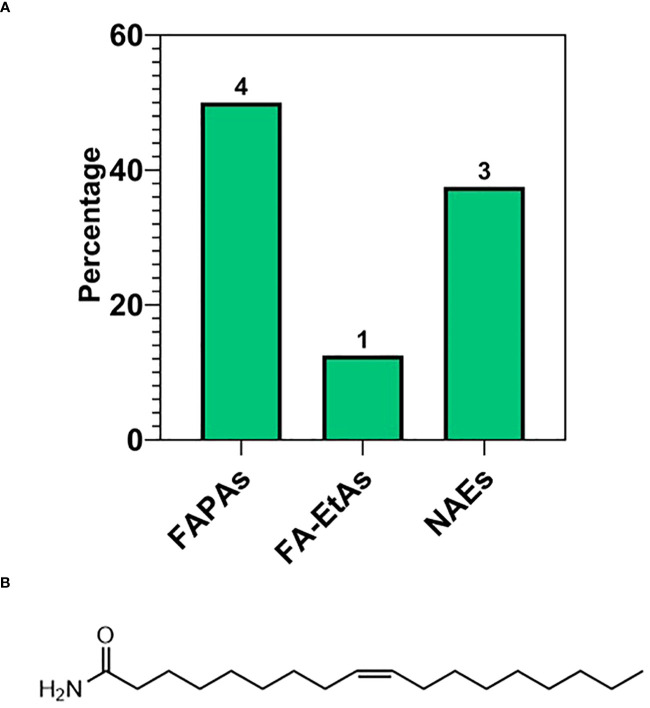
**(A)** Subspecies of fatty acid amides (FAA): primary (FAPAs), ethylamide (FA-EtAs), N-acylethanolamines (NAEs). **(B)** Molecular structure of the most abundant FAA reported in ‘**Table 3**’, namely Oleamide.

The third most abundant subclass of fatty acids was the fatty acid amides (FAAs). FAAs were classified as fatty acid primary amides (FAPAs), N-acylethanolamines (NAEs), and ethylamides (FA-EtAs), which accounted for 50% (4), 37% (3), and 12.50% (1) of the total FAA profile, respectively (**Figure 8A**). The identified FAAs possessed chain lengths of C16 and C18, including palmitoyl ethanolamide, palmitamide, stearamide, oleoylethanolamide, and oleamide. Erucamide was the only FAA with a C22 carbon chain. The list of these species and their relative abundances (%) are reported in **Table 3**, with their molecular structures shown in **Figure 8B**.

**Table 3:** Fatty acid amides (FAAs) with their names, m/z, type of ionization and relative abundance (%) calculated as explained in section 2.3 of Material and Methods.

Metabolite Name	m/z	Ionization	Relative Abundance %
Stearamide	284.2946	[M+H] +	3.31
Palmitoleoyl ethanolamide	280.2632	[M+H-H_2_O] +	2.82
Palmitamide	256.2633	[M+H] +	8.29
Oleoyl ethylamide	310.3102	[M+H] +	1.12
Oleamide	282.2789	[M+H] +	55.31
N-Oleoylethanolamine	326.3051	[M+H] +	5.89
Linoleoyl ethanolamide	324.2894	[M+H] +	7.42
Erucamide	338.3415	[M+H] +	15.84

### 3.4 Quantification of oxylipins and NAEs in waste by-products enriched in walnut pellicle

After their detection in the untargeted metabolomics analysis, oxylipins and N-acylethanolamines (NAEs) were quantified in two industrial waste streams from handling processes of walnuts in California, named “Sorting Meal Room (SRM)” and “Blower Fluff (BF)”. While not the only walnut processing waste streams, SRM and BL were selected for being enriched in pellicle and generally considered as abundant, renewable, and low-value agricultural waste. These wastes were of particular interest for potential re-utilization and valorization into high-value products due to their array of potent bioactive lipids relevant to plant and human health.

**Figure 9:**
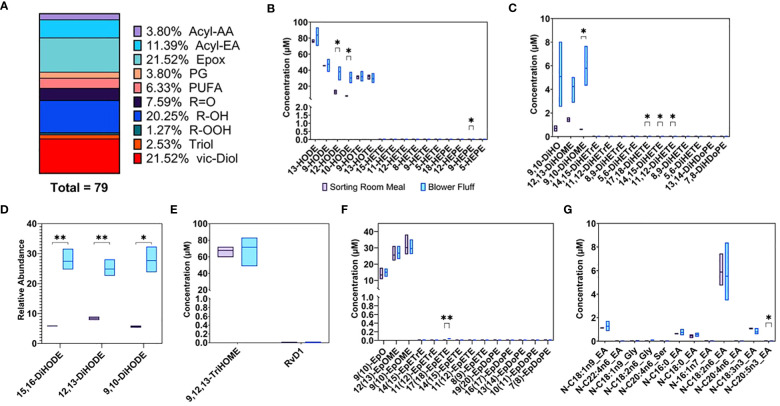
**(A)** % Subclasses of Oxylipins and endocannabinoid compounds detected and quantified by targeted analysis in waste byproducts. Concentrations expressed as μM of **(B)** Monohydroxy **(C)** Vicinal dihydroxy **(E)** Trihydroxy **(F)** Epoxy **(G)** Amide fatty acids. **(D)** Vicinal hydroxy fatty acids expressed as relative abundance. * p-value < 0.05, ** p-value < 0.01.

Oxylipins derived from free fatty acids and NAEs, also called Acyl-ethanolamides (Acyl-EAs), were quantified in SRM and BF wastes along with ketones, PUFAs, Acyl-Amino Acids (Acyl-AAs), and prostaglandins (PGs). The 74-member panel of metabolites was identified and quantified by targeted metabolomic. As for percentages, oxylipins composed 74.68% of the panel, followed by Acyl-EAs (11.39%), PUFAs (6.33%), Acyl-AAs, and PGs (3.8%). Within the oxylipins group, metabolites were distinguished by oxygenation type (**Figure 9A**): including epoxides of fatty acids (21.52%), monohydroxy fatty acids (R-OH) (20.25%), vicinal diols (vic-Diols) (21.52%), ketones (R=O) (7.59%), triol fatty acids (2.53%), and hydroperoxy fatty acids (R-OOH) (1.27%).

Concentrations of monohydroxy fatty acids, including 13-, 9-, 12-, and 10-HODE, were the highest along with triols, like 9,12,13-TriHOME (**Figures 9B, E**), in both waste streams, reaching concentrations of 80–100 μM. Comparing the two waste streams, BF was more enriched in vicinal diols like 15,16-, 12,13-, and 9,10-DiHODE (**Figure 9D**). These subsets of metabolites are reported as relative abundance across all measured samples (i.e., the sum of each metabolite across all samples is set to 100%) rather than concentrations in μM. Along similar lines, 9,10-DiHO, as well as 12,13- and 9,19-DiHOME, were also more abundant in BF and had lower concentration compared to triols and R-OH (**Figure 9C**). Epoxides of fatty acids were enriched in both wastes: 9(10)EpO, 12(13)EpOME, and 9(10)EpOME were detected in concentrations of 10–35 μM (**Figure 9F**).

A rich profile of NAEs was detected and quantified (**Figure 9G**). Linoleoylethanolamide (N-C18:2n6_EA) was the most abundant NAE detected in both waste streams with an average value of 5.7 μM, followed by oleoylethanolamide (N-C18:1n9_EA) at 1.2 μM, linolenoylethanolamide (N-C18:3N3_EA) at 0.9 μM, palmitoylethanolamide (N-C16:0_EA) at 0.69 μM, and finally stearoylethanolamide (N-C18:0_EA) at 0.5 μM. The complete list of metabolites detected and quantified in both waste by-products is included in **Supplementary Table 5**.

## 4 Discussion

This study is the first deep metabolomic exploration of the lipid profile of the walnut pellicle. The various analyses brought to light many unusual lipid species, particularly fatty acids, possessing specific bioactivities. For the plant, these compounds contribute to the barrier function of the pellicle, and for mammals, the same compounds serve diverse physiological roles. These discoveries spurred the detection and quantification of a number of these compounds in the pellicle-enriched waste streams of the California walnut industry. The discovery of significant concentrations of these bioactive lipid species revealed these classically low-value waste byproducts to be untapped sources of bioactive lipids, paving the way for waste reutilization and valorization.

### 4.1 Comparison of lipid compositional profile between the walnut pellicle and kernel

Establishing a successful process for pellicle isolation from nutmeat enabled the identification of metabolism specifically occurring in the pellicle. An untargeted metabolomic analysis enabled a comprehensive qualitative description of pellicle lipid composition. The proportion and composition of major lipid classes, expressed as percentages of total feature counts, were broadly consistent between isolated pellicles and previously studied kernels (including the nutmeat and the pellicle in most published studies), although with interesting differences.

Free fatty acid (FFA) species were also found to represent a considerable portion of the pellicle lipid population. Shown extensively in the literature, walnut kernels possess a high content of polyunsaturated fatty acids (PUFAs), especially linoleic acid, and the lowest saturated fatty acids: total fatty acids ratio and greatest content of PUFAs compared with other nuts (Vecka et al., 2019; Jardim et al., 2023). Accumulation of long-chain (LC) FFAs like linoleic, oleic, and linolenic acids has also been shown in dried walnut shells and husk tissue (Ventura et al., 2023; Herrera et al., 2020; Romano et al., 2021; Barekat et al., 2023). In walnut seed coat, LC-PUFAs, LC-MUFAs and LC-SFAs, including their oxygenated derivatives called oxylipins, accounted for 40.91% (18), 22.72% (10), and 6.82% (3), respectively of the total FFA counts (44 features) and roughly 5%, 3% and 1% of the total identified lipid features (318 features). In general, lipidomic profile of walnut kernel is more enriched, both quantitatively and qualitatively, in unsaturated fatty acids than saturated (Yan et al., 2021; Okatan et al., 2022; Jardim et al., 2023; Zhang R. et al., 2021), a trend recently shown to be conserved across several walnut genotypes (Okatan et al., 2022). Our study provides qualitative results which support previous research; indeed, similarly to kernel FFA composition, the majority of the FFAs detected in walnut seed coat were LC-PUFAs and LC-MUFAs, summing up to the 63.7% of the total FFA features’ count. More broadly, **Figure 5B** shows distribution of degree of unsaturation in the entire class of FFAs detected in our data, including very long-, long- and, medium-chain fatty acids, as well as oxygenated and non-oxygenated FFAs. This confirms that polyunsaturation is the most prevalent feature of FFAs class in walnut seed coat.

The majority of LC-MUFA and LC-PUFA features detected in walnut pellicle were oxygenated in the form of hydroxy or epoxy long-chain fatty acids (**Figure 5A**). While non-oxygenated LC-MUFAs and LC-PUFAs represented just the 9% each of the total FFAs, oxygenated derivatives of LC-MUFAs and LC-PUFAs (shown in **Figure 5A**) reached 13.64% (6) and 31.82% (14) of the total FFAs, respectively. These results highlighted the accumulation of oxylipin derivatives of conventional fatty acids, like linoleic and linolenic acids, in walnut seedcoat, therefore suggesting that levels of oxygenated LC-MUFAs and LC-PUFAs might differ between nutmeat and seedcoat. To the best of our knowledge, no studies have been published showing LC-MUFA and/or LC-PUFA quantification specifically in walnut seed coat, as well as the quantification of their oxylipin derivatives in kernel tissues. Nevertheless, considering together the numerous lipidomic studies on the topic and our results, we could not rule out the possibility that concentrations of LC-MUFAs and LC-PUFAs would be more enriched in nutmeat than in the seed coat, where those fatty acids might be preferentially metabolized into their oxylipins derivatives. This hypothesis is valuable in light of the biological function of oxylipins in walnut seed-coat as protection against stress for the nutmeat as explained in section 4.3.1. Targeted quantitative analyses of those fatty acids in both seedcoat and nutmeat is needed to further validate this hypothesis.

Also revealed in this study was the presence of very long chain (VLC) FAs, with carbon chain lengths containing 22–30 carbon atoms, which are rare and in low abundance within the kernel (Yan et al., 2021; Jardim et al., 2023). These species accounted for 18.18% (8) of the total FFA counts in the pellicle (**Figures 5A, C**). As a minor class of the FFAs, medium chain (MC) FAs were identified at similar abundance to the kernel (Yan et al., 2021), making up 11.36% of the FFA counts (**Figures 5A, C**). Overall, this analysis revealed the pellicle FFA profile to be compositionally distinct from the reported kernel FFA profile, with the pellicle containing many uncommon metabolites to be discussed later (**Figures 4C**, **6C**).

The reported walnut kernel lipid profile generally places glycerolipids (GLs) as the predominant lipid class which was mirrored in the pellicle, with triacylglycerols (TGs) being the most abundant subclass followed by diacylglycerols (DGs) and monoacylglycerols (MGs) (Yan et al., 2021; Zhang R. et al., 2021; Wang et al., 2022). Interestingly, galactolipids falling under the larger DG class were detected and consistent with prior reporting. These include digalactosyldiacylglycerol (DGDG), monogalactosyldiacylglycerol (MGDG), and sulfoquinovosyl diacylglycerol (SQDG), with DGDG and MGDG accounting for less than the 5% and 2% of the total GLs and lipid classes respectively (Yan et al., 2021; Zhang R. et al., 2021). The levels of these compounds are influenced by drying and may be accumulated as the pellicle matures and desiccates, as well as from commercial drying processes (Wang et al., 2022).

This agreement between pellicle and kernel composition was largely the case for phospholipids (PLs), sphingolipids (SLs), and terpenoids/isoprenoids. Concerning the PLs, PCs were the most accounted for subclass, followed by phosphatidylinositols (PIs), phosphatidylethanols (PEs), and phosphatidic acids (PAs) (Yan et al., 2021; Zhang R. et al., 2021; Wang et al., 2022; Jardim et al., 2023). Furthermore, high frequency of lyso-PCs, up to 24% (Yan et al., 2021) and 26% (Zhang R. et al., 2021) of the total PC population and 5% (Yan et al., 2021) and 6% (Zhang R. et al., 2021) of the total PL population, was found in the walnut kernel in other studies. These species, which arise from enzymatically-mediated PC hydrolysis by the action of phospholipases, like PLA2, can be highly dependent on genotype, tissue maturity, and abiotic stresses imposed by management practices such as drying (Wang et al., 2022) and storage conditions (Zhang R. et al., 2021). Regarding SLs, the walnut kernel and isolated pellicle are more enriched in simple sphingolipids like ceramides than complex sphingolipids like HexCer. The SL population makes up roughly 10% of the total lipid metabolites for the pellicle, similar to the case of the kernel (Yan et al., 2021; Zhang R. et al., 2021). Similar to the PLs, the increase in SLs was reported in the kernel to be an outcome of the drying process (Wang et al., 2022). As for terpenoids/isoprenoid class, phytosterols (i.e., steroidal alcohols) belonging to the group of triterpenoids were mainly found in nuts, including whole walnuts (Jardim et al., 2023) and walnut oil (Rébufa et al., 2022), while a more complex profile for other terpenoid subclasses was found for walnut leaves by VOC analysis (Valdés García et al., 2021). Unlike the kernel, the pellicle was revealed to possess a more diverse repertoire of terpenoid subclasses, including mono-, di-, tetra-, and sesquiterpenoids (in addition to the phytosterols as mentioned above). While monoterpenoids were previously identified in walnut kernels by supercritical fluid extraction coupled to GC-MS analysis, detection of further subclasses was not reported (Kessler et al., 2023). Overall, these results are broadly consistent with the published data for the walnut kernel (Huang et al., 2021; Yan et al., 2021; Zhang L. et al., 2021; Zhang R. et al., 2021; Wang et al., 2022) obtained with analytical pipeline similar to one adopted for this study.

A summary of the lipid compositional profile of walnut tissues reported in most recent publications is presented in **Table 4**.

**Table 4:** Lipid and fatty acid classes detected in walnut tissues and their related bioactivity in human and plants; publications cover years 2019–2024.

Lipid and fatty Acid Class	Walnut Tissue	References on detection of lipid and fatty acid classes in walnut (time range 2019–2024)	Bioactivity of lipid and fatty acids classes detected in walnuts in human and plant
MG, DG, TG	Whole kernel	(Yan et al., 2021) (Wang et al., 2022) (Zhang R. et al., 2021)	Major form of stored energy in animals and plants (Yang and Benning, 2018; Zadoorian et al., 2023)
PA, PC, PE, PG, PI, PS, including Lyso- forms, MePC, PMe	Whole kernel	(Yan et al., 2021) (Zhang R. et al., 2021) (Wang et al., 2022)	Cell Membrane Structure and Function, Mitochondrial function and energy production, regulation of brain and gut health (Cai and Yang, 2018; Chen et al., 2019; Dai et al., 2021). Cell membrane structure, regulatory and signaling functions (Nakamura, 2017)
DGMG, MGDG	Whole kernel^a^ Shells^b^	(Yan et al., 2021)^a^ (Zhang R. et al., 2021)^a^ (Wang et al., 2022)^a^ (Herrera et al., 2020)^b^	Cell Membrane Structure and Function, anti-inflammatory, anti-tumor, regulation of immune system and neurological function (Christensen, 2009). Component of plant photosynthetic membranes (Bolik et al., 2022)
SQDG	Whole kernel	(Yan et al., 2021)	Component of plant photosynthetic membranes (Bolik et al., 2022)
Cer, HexCer, SL, SM	Whole kernel	(Yan et al., 2021) (Zhang R. et al., 2021) (Wang et al., 2022)	Neuroprotection, regulation of intestinal immunity, mediation of cancer development, skin protection, regulation of metabolic dysfunctions and apoptosis in humans (Wang X. et al., 2021). Cell membranes structure, Signaling, response to biotic and abiotic stress, regulation of apoptosis in plants (Michaelson et al., 2016)
Fatty acyls (in particular unsaturated fatty acids)	Whole kernel^a^ Shells^b^ Husk^c^	(Vecka et al., 2019)^a^ (Yan et al., 2021)^a^ (Zhang R. et al., 2021)^a^ (Wang et al., 2022)^a^ (Okatan et al., 2022)^a^ (Herrera et al., 2020)^b^ (Ventura et al., 2023)^b^ (Romano et al., 2021)^c^ (Barekat et al., 2023)^c^	Reduction of low-density-lipoprotein cholesterol, energy homeostasis through PPAR-alpha, provides protective and stabilizing effects of cellular membranes, anti-inflammatory effect though activation of PPAR-gamma (Hooper et al., 2020; Ramsden et al., 2021; Lockyer et al., 2022; Kim et al., 2023). Component of TAGs for energy reserve, precursors of extracellular barrier constituents (e.g., cutin and suberin), precursors of bioactive molecules (e.g., jasmonates), and regulators of stress signaling (He and Ding, 2020).
Hydroxy Fatty Acids	Shells	(Herrera et al., 2020)	Reviewed in sections 4.3.1 for plant bioactivity and 4.4.1 for human health
NAE	Whole kernel	(Wang et al., 2022) (De Luca et al., 2019)	Reviewed in sections 4.3.3 for plant bioactivity and 4.4.3 for human health
Sterols	Whole kernel^a^ Shells^b^ Husk^c^	(Vecka et al., 2019)^a^ (Wang et al., 2022)^a^ (Herrera et al., 2020)^b^ (Barekat et al., 2023)^c^	Cardioprotective, neuroprotective properties, anti-aging, skin regeneration effect (Kopylov et al., 2021). Components of membranes, regulation of development, role in defense against biotic and abiotic stress in plants (Rogowska and Szakiel, 2020)
Prenols (Terpenoids)	Whole kernel^a^ Shells^b^ Husk^c^	(Wang et al., 2022)^a^ (Jardim et al., 2023)^a^ (Kessler et al., 2023)^a^ (Herrera et al., 2020)^b^ (Romano et al., 2021)^c^ (Barekat et al., 2023)^c^	Anticancer, antimicrobial, anti-inflammatory, antioxidants, antiallergic, neuroprotective and anti-coagulation activity, sedative and analgesic properties in human (Masyita et al., 2022). Regulation of development, signaling, role in defense against biotic and abiotic stress in plants (Tholl, 2015)
VOCs (i.e., alcohols, esters, aldehydes, acids, and ketones)	Whole kernel^a^ Husk^c^	(Okatan et al., 2022)^a^ (Kessler et al., 2023)^a^ (Romano et al., 2021)^c^ (Barekat et al., 2023)^c^	Mediate the interaction of plants with pollinators, herbivores, other plants and micro‐organisms; role in plant defense (Bouwmeester et al., 2019). Anti-inflammatory, support immune system, ameliorate mood (Antonelli et al., 2020)

MG, Monoacylglycerol; DG, Diacylglycerol; TG, Triacylglycerol; PA, Phosphatidic Acid; PC, Phosphatidylcholine; PE, Phosphatidylethanolamine; PG, Phosphatidylglycerol; PI, Phosphatidylinositol; PS, Phosphatidylserine; NAE, N-Acylethanolamine; MePC, Methyl phosphatidylcholine; PMe, Phosphatidylmethanol; DGDG, Digalactosyldiacylglycerol; MGDG, Monogalactosyldiacylglycerol; SQDG, Sulfoquinovosyl diacylglycerol; Cer, Ceramide; HexCer, Hexosylceramide; SL, Sphingolipid; SM, Sphingomyelin. Superscript letters are used to indicate the references reported in the same table and related to papers showing analysis of each walnut tissue. This is useful when there are more than one tissue.

### 4.2 Characterization of unusual free fatty acids in the walnut pellicle

As previously mentioned, fatty acid species comprised 21.70% of the total lipids detected in the walnut pellicle, more than twice the number detected in the kernel by Yan et al. (Yan et al., 2021) and Wang et al. (Wang et al., 2022). The FFAs as a subclass constituted 63.77% of the FA profile, and similar to the kernel (Yan et al., 2021; Wang et al., 2022), were primarily LC-MUFAs and LC-PUFAs (**Figures 5A–C**). However, these groups contained many unusual members aside from the conventional FAs (i.e., C18:1 oleic, C18:2 linoleic, and C18:3 linoleic acid) in the pellicle. Half of the total FFAs (50%) were oxygenated in the form of hydroxy-, peroxy-, or epoxy-fatty acids, which fall into the umbrella term of “oxylipins” (**Figures 5D**, **6B**). These FFA-derived oxylipins are also involved in the production of related molecular families, including jasmonic acid (Acosta et al., 2009) and certain γ-lactones that may contribute to the barrier function of the pellicle. Independent of the oxylipins, the second and third significant subclasses of detected FFAs were the dicarboxylic FAs (DC-FAs) and the fatty acids amides (FAAs). To our knowledge, this is the first reported detection of DC-FA in walnut tissue, as prior metabolomic studies of walnuts did not report this unique subclass of FFAs. Regarding the FAAs, of which N-acylethanolamines (NAEs) are a subgroup, detection and quantification of NAEs in the whole walnut kernel have been reported previously (De Luca et al., 2019).

### 4.3 The roles of unusual free fatty acids as mediators of critical pellicle physiological processes

#### 4.3.1 Oxylipins

In general, oxylipins have been implicated in various critical functions related to plant defense. The species identified within the walnut pellicle were predominantly long-chain hydroxy FAs, a central component of cutin. This insoluble lipophilic polyester makes up the plant cuticle framework and intracuticular waxes made of VLC-FAs like those detected in this study. This waxy layer on the outer surface of the plant epidermis creates a hydrophobic skin around plant tissue, controlling the exchange of moisture and gasses with the environment, as well as providing protection against would-be pathogens (Fich et al., 2016; Cohen et al., 2017). Interestingly, monomers of this lipophilic polyester, i.e., hydroxy fatty acids, were found in walnut seedcoat as also in walnut shells (Herrera et al., 2020). Degradation of the cuticle by pathogen-secreted cutinases produces bioactive oxylipins, namely monohydroxy-, polyhydroxy-, and epoxy FAs, that serve as damage-associated molecular patterns (DAMPs) that trigger the plant immune response. These same compounds also act directly as antimicrobials, with several hydroxy FAs known to possess antifungal properties (Pohl et al., 2011; Guimarães and Venâncio, 2022). Among the oxylipins detected in the pellicle and further validated by quantification in pellicle-enriched waste streams, the trihydroxy fatty acid *9,12,13-TriHOME* have demonstrated antifungal activity against black rot fungus *(Ceratocystis fimbriata);* other isomers of trihydroxy fatty acids also showed antifungal activity against aggressive oomycete pathogens like *Phytophthora* (Pohl et al., 2011). Trihydroxy fatty acids (like 9,12,13-TriHOME) are derivatives of hydro-epoxy fatty acids; where the epoxy group was hydrolyzed by the action of plant soluble epoxide hydrolase (sEH). Similar to mammals, plant sEHs are mainly present in the cytosol, and their activity is associated with growth processes and stress. Indeed, plant sEHs are involved in defense mechanisms supporting the synthesis of cutin monomers and antifungal compounds like the aforementioned trihydroxy fatty acids (Newman et al., 2005). The monohydroxy fatty acids 9- and 13-OH C18:3 is also known to have an antifungal effect against *Alternaria* (Pohl et al., 2011), which can cause mold in walnut kernels. Similar antifungal activity has been observed in saturated and unsaturated FAs (i.e., palmitic and linoleic acids) and other mono- and di-hydroxy FAs against *Fusarium* and *Phomopsis* that cause molding in walnuts (Pohl et al., 2011; Tournas et al., 2015). From an evolutionary standpoint, the development of a waxy layer with potent antimicrobial properties can be considered an enabling structure for the colonization of land by plants and expansion into increasingly harsh environments (Fich et al., 2016). This is all the more critical for seed coats, like the walnut pellicle, that serve the essential role of protecting a plant’s progeny against abiotic and biotic stressors that could compromise survivability. The detection of VLC-FAs and oxylipins in the pellicle is evidence of the wax and cutin layers, respectively, which protect the susceptible oil-rich seed from abiotic and biotic stress.

Aside from being produced as part of the cuticular barrier, oxylipins accumulate in plant tissues through two other noteworthy pathways: synthesis within oil bodies (OBs) and intracellular oxygenation of membrane-derived PUFAs. OBs (i.e., lipid micelles) function as subcellular factories for antifungal oxylipins (Shimada and Hara-Nishimura, 2015; Cahoon and Li-Beisson, 2020) and are enriched in FA-containing TGs and oxylipin biosynthetic enzymes like phospholipases (PLA2), α-dioxygenases (α-DOX), and 9- and 13-lipoxygenases (LOX-9 and LOX-13) (Shimada and Hara-Nishimura, 2015). OBs accumulate in plant embryos and oil-rich seeds of some plant species such as *Vernonia galamensis and Stokesia laevis* (Li et al., 2010), as well as in senescent plant tissues where lipids are actively recycled from membranes and organelles. In senescent tissues, these OBs serve as peroxisomal energy sources for succinate production to fuel gluconeogenesis (Shimada et al., 2018). As the walnut pellicle achieves maturity, it enters senescence. It is likely consequently enriched in OBs, which is consistent with the molecular hallmarks of this process (like 9- and 13- monohydroxy fatty acids and abundant TGs) observed in this study. Comparative proteomic analysis of walnut seed coat tissue across maturity stages showed the accumulation of proteins crucial for OB synthesis and defense activity; specifically, oleosin and oil body-associated proteins, along with lipoxygenases (Zaini et al., 2020). These previous results, together with this current study, support the hypothesis that OBs participate in oxylipin synthesis in the walnut seed coat. Another route leading to the accumulation of oxylipins is intracellular PUFA oxygenation following the liberation of FFAs from membrane lipids. This conserved cellular metabolism is fundamental to inducing signaling cascades and immune responses, like in the case of jasmonic acid, where the initial synthesis starts from the oxygenation of liberated linolenic acid (Dye et al., 2020; He and Ding, 2020). As tissue senescence is an oxidative stress-driven process, this may activate the molecular mechanisms for defense, such as membrane-derived PUFA oxidation for bolstering cellular chemical defenses.

#### 4.3.2 Dicarboxylic fatty acids

Revealed in this study was an unexpected presence of DC-FAs. This family of FFAs is produced in plants and mammals through ω-oxidation, an alternative lipid catabolism route in the endoplasmic reticulum and microsomal membranes (Mingrone and Castagneto, 2006; Compagnon et al., 2009). This family may be considered hallmarks of stress and mitochondrial dysfunction, as ω-oxidation is the “rescue plan” when β-oxidation is compromised (Miura, 2013). The produced DC-FAs may be catabolized in the peroxisome to maintain energy homeostasis and transferred out of the cell along with other oxylipins as precursors to the biopolymers cutin and suberin (Kolattukudy, 2001; Fich et al., 2016), and serve as intracellular molecular signals to induced defense responses. In plants, this occurs in various organs under particular conditions, such as geminating cotyledons and senescing leaves (Mingrone and Castagneto, 2006), while in mammals, the production of DC-FAs is associated with starvation and severe metabolic dysfunction. Among the DC-FAs identified in the pellicle, azelaic acid is an essential regulator of plant immune response. Stress-induced production of reactive oxygen species (ROS), which serve as mediators for rapid cellular signaling in a cascade, leads to breakage and oxidation of plastid FAs into azelaic acid. The azelaic acid, in turn, primes the immune response through the induction of SAR-inducer glycerol 3 phosphate (Wang C. et al., 2014) and salicylic acid (Jung et al., 2009). With regard to the pellicle, oxidative stress is imposed during development, maturation, and desiccation (Kurek et al., 2019). Taken together, the synthesis of DC-FAs might be considered a molecular biosignature of mitochondrial dysfunction and cell energy impairment during pellicle senescence and desiccation.

#### 4.3.3 Fatty acid amide

Maturation and senescence processes in seeds are marked by significant oxidative stress and signaling by abcisic acid (ABA) in preparation for desiccation (Zhang et al., 2005; Kurek et al., 2019). ROS and ABA induce the expression of phospholipase D (PLD) as a regulator of crucial signaling networks during these programmed cellular processes and biotic and abiotic stress (Chapman, 1998; Zhang et al., 2005; Chen et al., 2018). PLD is responsible for hydrolyzing N-acylphosphatydilethanolamines (NAPEs), a subset of the PLs located in cellular membranes, leading to the synthesis of NAEs (Blancaflor et al., 2014). These NAEs are negative molecular regulators of plant growth by inhibiting plastid development and function (Arias Gaguancela and Chapman, 2022). Inhibition of plastid function is detrimental to energy homeostasis due to its complex relationship with the mitochondria (mediated by various signals, including ROS). Down-regulation or functional impairment of either of these two organelles can lead to extensive cellular damage and even to programmed cell death (PCD) in a cooperative manner (Van Aken and Van Breusegem, 2015). Indeed, typical plastids in nuts are called elaioplast and are crucial for synthesizing fatty acids used as energy substrates by mitochondria (Choi et al., 2021). Another molecular sign of mitochondrial stress is the presence of fatty acid primary amides (FAPAs), like oleamide, which have been detected in this study. In mammals, oleamide can modulate intracellular calcium (Ca2+) levels, which causes mitochondrial stress and the release of cytochrome-c, triggering PCD mediated by apoptosis (Mueller and Driscoll, 2009). A similar function can be hypothesized to occur in the case of plants. NAEs-induced stress in plastids can trigger caspase-like activity following the release of cytochrome-f from plastids (Samuilov et al., 2003; Wang H. et al., 2014; Ambastha et al., 2015). Senescence-dependent PCD driven by the down-regulation of plastid function and mitochondrional apoptosis is a plausible mechanism of organelle disruption during senescence and desiccation processes (Del Duca et al., 2014; Mayta et al., 2019). This is supported by the detection of ceramides in this study, SLs that are known regulators of PCD. Also, previous research in our group detected significant changes in cytochrome c expression, activation of several metacaspases, and enrichment of peptidase activity in the walnut seed coat during nut maturation (Zaini et al., 2020). Those previous results, along with the detection of FAPAs, like Oleamide, in this study, reinforce the hypothesis of apoptosis-driven PCD during maturation; ultimately leading to seed coat senescence and desiccation. Given that the walnut pellicle at the late stages of maturity is dead tissue, as evidenced by the distinct disappearance of organelles, the release of NAEs may be a regulated process to interrupt maturation and shift into senescence and desiccation (Wu et al., 2009).

**Figure 10:**
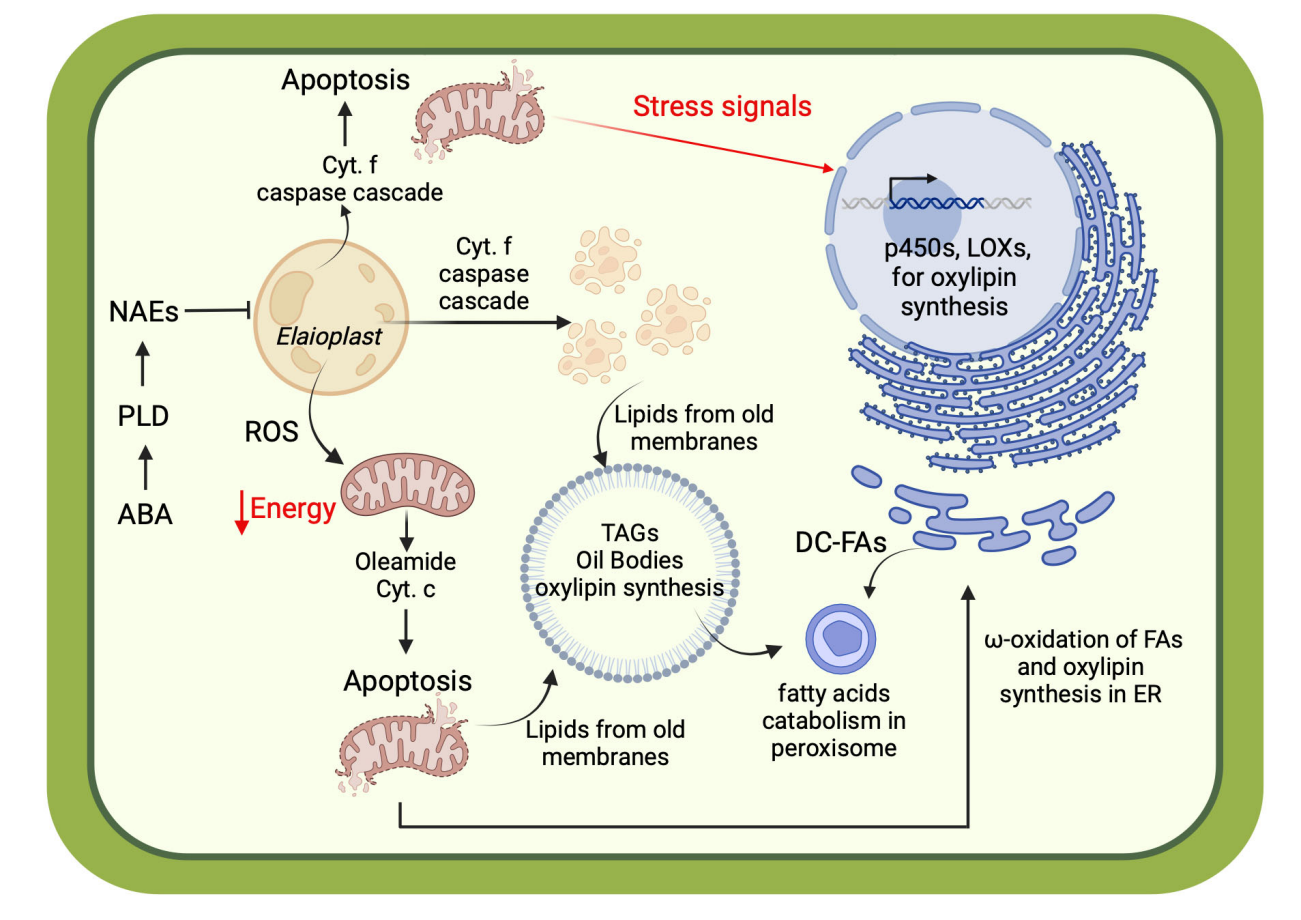
Cellular scheme describing the molecular regulation of senescence mediated by bioactive lipids: abscisic acids (ABA); phospholipase of type D (PLD); N-acylethanoamines (NAEs); endoplasmic reticulum (ER); lipoxygenases (LOXs). Created with BioRender.com.

Overall, these various classes of bioactive lipids identified in this study provide an elegant explanation of the molecular processes intrinsically related to stress resilience and the physiological processes associated with the programmed desiccation of the walnut pellicle (**Figure 10**).

### 4.4 Bioactive lipids: a connection between plant and mammalian health

Intriguingly, the same classes of bioactive lipids identified in this study show potent bioactivities in mammals (notably humans) and are pivotal modulators of various physiological processes related to health and wellbeing. As previously mentioned, bioactive lipids can have peculiar structural features, including oxygenations of the carbon chain, conjugation to other bioactive molecules (like amino acids), or unusual degree of unsaturation (Cahoon and Li-Beisson, 2020).

#### 4.4.1 Oxylipins

Oxylipins are bioactive lipids resulting from the oxygenation of PUFAs. This is a highly conserved molecular pathway among plants and mammals, as oxylipins are the backbone of numerous signaling molecules that function as homeostatic regulators for immunity, inflammation, and other physiological processes preventing serious diseases. In mammals, ω6- and ω3-PUFA metabolism is mediated by the activity of cyclooxygenases and lipoxygenases, producing both pro- and anti-inflammatory oxylipins, respectively (Leuti et al., 2020). Additionally, cytochrome P450 (CYP450) enzymes mediate the epoxidation of PUFAs at existing unsaturations along with omega and omega-1 hydroxylations (Sarparast et al., 2020). The omega-hydroxy- and epoxy-PUFAs interact to modulate pain and inflammation, the functions of the immune and cardiovascular systems, skin barrier functions, and various metabolic dysfunctions (like obesity) (Imig, 2016; Elshenawy et al., 2017; McReynolds et al., 2020; Zhang et al., 2020). A notable role of the CYP450s is the epoxidation of endocannabinoids, like anandamide, identified in the brain, heart, and liver (McDougle et al., 2017). The epoxy fatty acids (EpFAs) compounds have essential bioactivities but can be easily hydrolyzed into their FA diols by sEHs; therefore, affecting their beneficial effect. Indeed, FA diols at low concentrations have been shown to improve lipid metabolism through increased fatty acid uptake in brown adipose tissue (BAT) and skeletal muscle. However, higher concentrations (above 100 μM) have shown proinflammatory activity and potential mediation of mitochondrial toxicity (Stanford et al., 2018; Zimmer et al., 2018; Hildreth et al., 2020). Epoxy fatty acids (EpFAs), like 9,10- and 12,13-EpOMe identified in walnut pellicle-enriched waste streams, are critical negative regulators of inflammation and pain. Aside from the EpFAs, this study identified multiple hydroxy FAs that are relevant to human health. This group of compounds exhibits antimicrobial properties, protecting against infections, and also serves as master regulators of energy metabolism, specifically through the enhancement of mitochondrial and peroxisomal FA metabolism by PPAR-α activation (Yokoi et al., 2010). This class of oxylipins is a promising therapeutic candidate for diseases driven by metabolic dysfunction. Hydroxy FA activation of PPAR-α in the brain has been shown to exert neuroprotective effects and improve memory (Liu et al., 2022), while derivatives of linoleic acid (like 9- and 13-HODE) are ligands for GPR132 (G2A) receptors in the skin, discouraging damaged cells from proliferating while stimulating the growth of keratinocytes (Kimura et al., 2020). As previously mentioned, several oxylipins detected in walnut seed coat exhibit antimicrobial activity, particularly antifungal properties, to protect the seed against pathogen invasion. Interestingly, walnut pellicle extracts (WPE) showed antimicrobial activity also against several human pathogens. Specifically, growth of *E. faecium*, *E. coli*, *K. pneumoniae*, and *P. aeruginosa* were inhibited by WPE (D’Angeli et al., 2021). The same WPE also demonstrated antifungal activity against multiple *C. albicans* strains, as well as inhibitory effects on HSV-1 and HSV-2 replication. Although WPE bioactivity was mainly attributed to bioactive polyphenol fraction, there is likely a contribution from the bioactive lipids, like oxylipins, to WPE’s antimicrobial activity. Considering the clinical outcomes from both EpFAs and hydroxy FAs, natural sources of these compounds for applications in health and wellbeing represent an exciting opportunity to improve quality of life. Walnuts have great potential to be a rich source of oxylipins; interestingly, walnut fermentation enriched the oxylipin profile (Fiorino et al., 2023). Quantification of both groups of metabolites in walnut industrial wastes was on the order of μM and can be consequently considered as good sources of potent oxylipins, specifically the Ep- and hydroxy-FAs.

#### 4.4.2 Dicarboxylic fatty acids

As previously mentioned, DC-FAs can rescue cellular energy metabolism through the oxidation route in case of mitochondrial impairment. Dysfunction of the mitochondria is a known underlying factor in many metabolic disorders, including increasingly prevalent conditions like diabetes. DC-FAs are essential substrates for cellular energetics as intermediates between FAs and sugars (like glucose). Their catabolism within the peroxisome produces succinic acid, feeding the TCA cycle and generating ATP in the case of mitochondrial dysfunction. This property is critically important during pathological conditions associated with insulin resistance and type-2 diabetes mellitus. For this reason, dietary supplementation with DC-FAs might be a vital tool for individuals who have diabetes. Previous studies showed that the administration of DC-FAs is safe and well tolerated in humans. However, more research is needed to understand the metabolism of DC-FAs within the context of different tissues and organs (Mingrone et al., 2013). Given this potential, natural sources of DC-FAs like the walnut pellicle can be used as inputs for food supplements and nutraceuticals targeted to individuals with diabetes or other metabolic conditions.

#### 4.4.3 Fatty acid amides

Lastly, this study revealed the presence of FAAs in the walnut pellicle and related wastes, particularly the N-acylethanolamines (NAEs), that considerably impact human health. The NAE family includes oleoylethanolamide (OEA), palmitoylethanolamide (PEA), stearoylethanolamide (SEA), and linoleoylethanolamide (LEA), among other possible analogues. NAEs are classified as para-endocannabinoids, as their structure resembles true endocannabinoids, like anandamide, but their bioactivity is not mediated by interaction with CB1 and CB2 cell surface receptors. Instead, the NAEs exert their action through the activation of nuclear receptors such as peroxisome proliferator-activated receptor alpha (PPAR-α), cell-surface transient receptor potential cation channel vanilloid-1 (TRPV1), and G-protein coupled receptor 119 (GPR119). Activation of GPR119 is especially noteworthy for its relationship with increasing satiety, as it increases GLP-1 secretion from intestinal L-cells, thus enhancing insulin levels and consequently inhibiting glucose-dependent glucagon secretion (Laleh et al., 2019). Further metabolic and pharmacological effects of NAEs supplementation include (but are not limited to) neuroprotection and anti-inflammatory action, as well as the amelioration of mood disorders, infertility, and the improvement of cognitive function (Ambrosini et al., 2006; Sun et al., 2007; Jin et al., 2015; Sayd et al., 2015; Yang et al., 2015; van Kooten et al., 2016; Antón et al., 2017; Joshi et al., 2018; Orio et al., 2019; González-Portilla et al., 2023). These outcomes suggest a profound role of NAEs as messengers of the gut-brain axis, being made or assimilated in the gut and traveling via the vagal nerve to stimulate the vagal sensory nerves by activating TRPV1 in the brain. Activation of TRPV1, which modulates pain and thermosensation, also contributes to regulating energy homeostasis by controlling feeding and energy expenditure (Laleh et al., 2019). Although endogenously produced in the human body (Schmid et al., 2002; Hu and Mackie, 2015; Herrera et al., 2016), primarily in the small intestine (Brown et al., 2017), NAEs can be readily degraded into FFAs and ethanolamine by intracellular FAA hydrolase (FAAH) and NAE-hydrolyzing acid amidase (NAAA) in the intestinal epithelium (Brown et al., 2017; Laleh et al., 2019). NAE catabolism is controlled through the interaction of genetic and external factors like stress, disease, and lifestyle that impact the presence and effects in the body. Inhibition of FAAH by a peculiar subclass of the endocannabinoids, the N-acyl amino acids (Acyl-AA) like N-acyl glycines (NAAs), play an essential role in the preservation of NAEs (Tegeder, 2016). Although in lower concentration than the NAEs, this study identified the Acyl-AAs within the walnut pellicle and waste byproducts. This makes walnuts a great source of NAEs (De Luca et al., 2019) and the FAAH-inhibiting Acyl-AAs. Taken together, this study revealed the walnut pellicle to be enriched in NAEs and the waste byproducts to be promising sources of this precious class of bioactive lipids, thus paving the way for the development of novel superfoods, food supplements, and nutraceuticals to regulate metabolic disorders (like diabetes and obesity), as well as improve cognitive health and energy homeostasis by way of the gut-brain axis.

### 4.5 Significance of the study

Considering the bioactive lipids’ relevance for human clinical outcomes, this broad group of compounds should be regarded as “essential bioactive dietary lipids”. Therefore, exploring edible dietary sources highly enriched in oxylipins, DC-FAs, and para-endocannabinoids (like NAEs and Acyl-AAs) is a promising avenue for improving the incorporation of these compounds into the human diet. Identifying these bioactive lipids in the walnut pellicle paves the way for identifying molecular features and pathways at the origin of the plethora of currently unexplained clinical outcomes (from cohort studies and randomized controlled trials) from walnut consumption. These outcomes range from reducing the risk of certain cancers, regulation of dysfunctional metabolic conditions like diabetes and obesity, improvement of cognitive function, amelioration of mood disorders and addiction behaviors, support of gut health by modulation of the gut microbiome, amelioration of male fertility, and age-related cognitive decline. While these conditions are major societal concerns, the latter are becoming increasingly important as the global population is aging and the fertility rate is declining in the industrialized countries. The consumption of functional foods to improve these various aspects of health and well-being is a significant trend among young adult consumers. For this reason, identifying bioactive lipids and their associated clinical outcomes might encourage walnut consumption in the younger population. Furthermore, the discovery of a natural source of bioactive lipids in the form of waste byproducts from walnut industrial processing will pave the way for developing novel edible supplements, nutraceuticals, or precursors for derma care products and pharmaceuticals. This discovery has significant economic impacts as rising walnut production will lead to a commensurate rise in waste by-products from the handling and processing pipelines. What have been classically low-value waste by-products may now be an opportunity for walnut industries to upcycle, encouraging sustainability and creating additional revenue streams to support industry longevity. Finally, walnut improvement programs may leverage breeding or bio-engineering approaches to enhance the production of these various bioactive lipids in the walnut pellicle or other walnut tissues to supply greater concentrations of these potent molecules for applications in human health.

## 5 Conclusion

This investigation described the deep lipid profile of the walnut pellicle for the first time. This has filled the previous knowledge gap of the molecular composition of this tissue, which has garnered increasing attention due to its high content of polyphenolic compounds related to quality and consumer health. We identified multiple classes of unusual fatty acids: oxylipins, dicarboxylic fatty acids (DC-FAs), and fatty acid amides (FAAs). These bioactive lipids possess pivotal bioactivities within the context of many plant physiological processes, including senescence, immunity, and stress amelioration. However, these same compounds possess potent bioactivities in humans, regulating various homeostatic and metabolic processes, highlighting their importance as dietary lipids. These bioactive lipids consequently represent the “hidden quality” of the walnut pellicle and pellicle-enriched waste streams. This work has recontextualized the potential value of pellicle-enriched waste streams from industrial walnut processing as unexpected, abundant, and renewable sources of these valuable lipids.

## Data availability statement

The original contributions presented in the study are included in the article/supplementary material, further inquiries can be directed to the corresponding author/s.

## Author contributions

RA: Formal analysis, Writing – review & editing, Conceptualization, Data curation, Formal analysis, Investigation, Methodology, Visualization, Writing – original draft, Writing – review & editing. NF: Conceptualization, Data curation, Formal analysis, Investigation, Methodology, Visualization, Writing – original draft, Writing – review & editing. IS: Data curation, Methodology, Writing – review & editing. JN: Data curation, Formal analysis, Methodology, Writing – review & editing. AD: Conceptualization, Funding acquisition, Project administration, Supervision, Writing – review & editing.

## Footnotes

1 U.S. Department of Agriculture and U.S. Department of Health and Human Services. Dietary Guidelines for Americans, 2020– 2025. (2020) https://www.dietaryguidelines.gov/sites/default/files/2020–12/Dietary_Guidelines_for_Americans_2020–2025.pdf. [Accessed March 3, 2024].

2 Walnut Market - Industry Analysis and Forecast (2023–2029). MAXIMIZE MARKET RESEARCH (blog). https://www.maximizemarketresearch.com/market-report/global-walnut-market/24016/ [Accessed February 29, 2024].

3 Qualified Health Claims: Letter of Enforcement Discretion - Walnuts and Coronary Heart Disease (Docket No 02P-0292), (2004)” Center for Food Safety and Applied Nutrition. http://wayback.archive-it.org/7993/20171114183725/ https://www.fda.gov/Food/IngredientsPackagingLabeling/LabelingNutrition/ucm072910.htm [Accessed February 29, 2024].

4 Walnut Related Health Claims | EFSA. (2020) https://www.efsa.europa.eu/en/efsajournal/pub/2074 [Accessed February 29, 2024].

5 National Weather Service, National Oceanic and Atmospheric Administration (NOAA). https://www.weather.gov/wrh/Climate?wfo=sto# [Accessed March 8, 2024].

## Supplementary material

Supplementary Figure 1 Separation of seed coat and nutmeat illustrated with a red-pigmented seed coat variety (*J. regia* cv. ‘Robert Livermore’) for clear visual distinction between the two tissues.

Supplementary Table 1 Raw data of the untargeted metabolomic profile from the analytical Platform 1.

Supplementary Table 2 Raw data of the untargeted metabolomic profile from the analytical Platform 2.

Supplementary Table 3 Raw data of the untargeted metabolomic profile from the analytical Platform 3.

Supplementary Table 4 Annotation of all metabolites detected and identified by the three analytical platforms.

Supplementary Table 5 Concentrations of oxylipins and endocannabinoids detected by targeted analysis in waste byproducts.

Supplementary Table 6 Targeted analysis’s additional information.

Supplementary Table 7 Relative abundances (%) calculated for all metabolites detected by the three analytical platforms.
