# Supplementary figures and images for: Unveiling the “hidden quality” of the walnut pellicle: a precious source of bioactive lipids
Source: Front Plant Sci. 2024 Jun 18;15:1395543. doi: 10.3389/fpls.2024.1395543 (PMC11217525; doi:10.3389/fpls.2024.1395543)

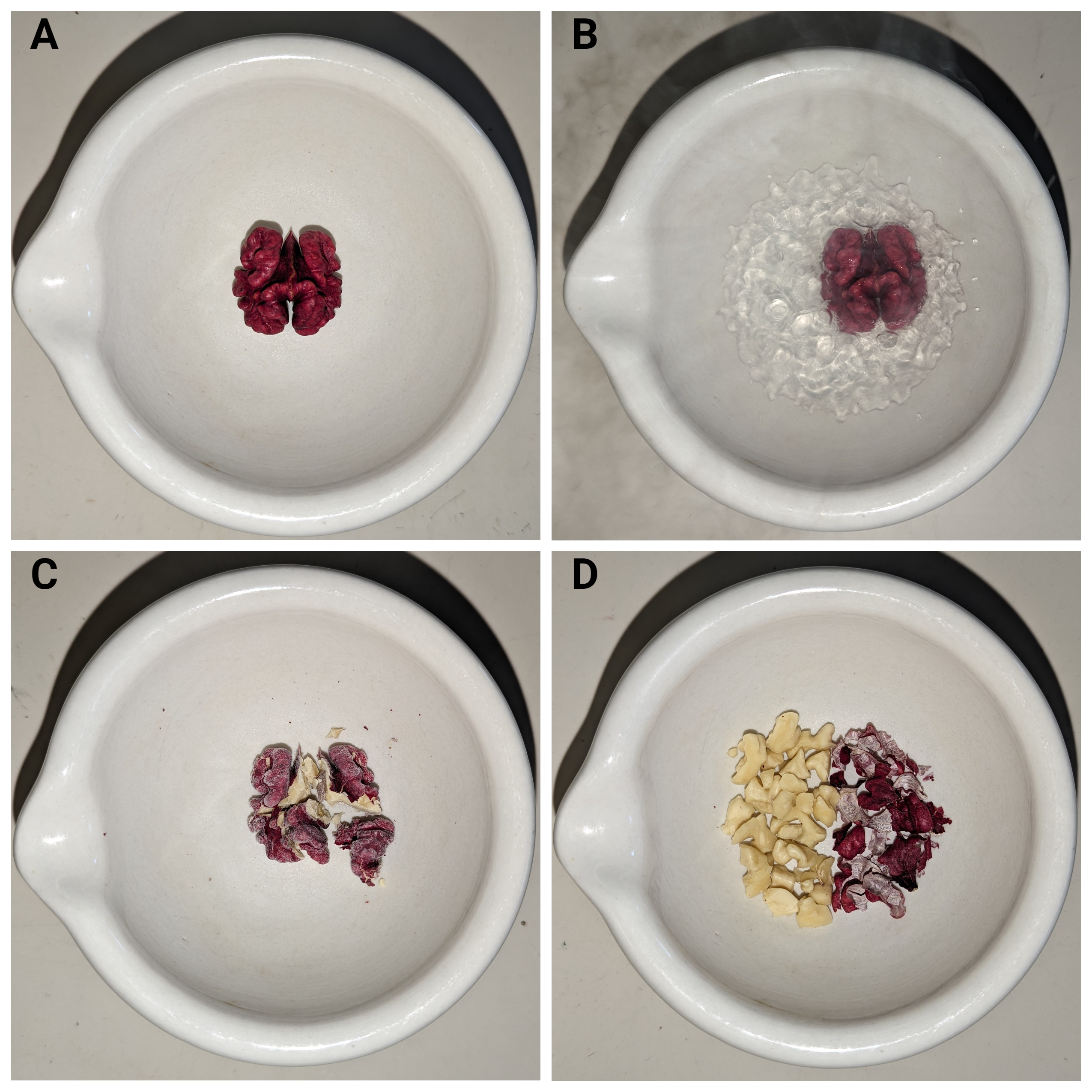

Supplement: Supplementary Figure 1 — Separation of seed coat and nutmeat illustrated with a red-pigmented seed coat variety (J. regia cv. ‘Robert Livermore’) for clear visual distinction between the two tissues. [file Image_1.jpeg]
